# Survey data of coronavirus (COVID-19) thought concern, employees' work performance, employees background, feeling about job, work motivation, job satisfaction, psychological state of mind and family commitment in two middle east countries

**DOI:** 10.1016/j.dib.2020.106661

**Published:** 2020-12-15

**Authors:** Mahfoudh Hussein Mgammal, Ebrahim Mohammed Al-Matari

**Affiliations:** College of Business-Jouf University & Amran University, Yemen

**Keywords:** Coronavirus (COVID-19), Work performance, Job satisfaction, Work motivation

## Abstract

The dataset presented in this article is an examination of coronavirus (COVID-19) thought concern, employees' work performance, employees background, feeling about job, work motivation, job satisfaction, psychological state of mind and family commitment in two middle east countries. The data were collected one time of survey data during June and July 2020 targeting people from different sectors whom have job through a survey distributed via an online questionnaire. Coronavirus (COVID-19) thought concern (9 items), employees' work performance (5 items), employees background (6 items), feeling about job (5 items), work motivation (6 items), job satisfaction (6 items), psychological state of mind (2 items) and family commitment (3 items). A mixture of purposive and snowball techniques facilitated to choose the respondents via email. We distributed 950 questionnaires through email due to the current circumstances, we offer suggestions from actual time-surveys handled in two Arabic countries the Yemen and Saudi Aribia, with a total final correct sample of 307 respondents. The survey data were analysed utilizing descriptive and inferential statistics. The data will assist in work place, companies and employees' awareness.

## Specifications Table

**Table:** 

Subject	Management, and Business.
Specific subject area	Management, Workplace
Type of data	Primary data,
How data were acquired	Data was gathered utilizing an online survey platform (google forms). The questionnaire is attached as a supplementary file.
Data format	Raw, combined, and filtered.
Parameters for data collection	The survey data was obtained through actual time-surveys handled in two Arabic countries the Yemen and Saudi Aribia, with a total final correct sample of 307 respondents.
Description of data collection	We collected a survey data on a geographically sample of people. A mixture of purposive and snowball techniques facilitated to choose the respondents via Email.
Data source location	Middle east, Arabic countries, Amran University, Yemen and Al-Jouf University, Saudi Aribia.
Data accessibility	Dataset is uploaded is and provided as a supplementary file

## Value of the Data

• The data has captured the moment thought of the employees’ group during the early part of the pandemic to record the anticipated rate of response in facing with such circumstances. The data is important in providing insights into coronavirus (COVID-19) thought concern, employees' work performance, employees background, feeling about job, work motivation, job satisfaction, psychological state of mind and family commitment in two middle east countries namely Saudi Arabia and Yemen.
• The data will be useful for researchers who want to compare with similar studies on COVID-19 thought concern and employees' work performance or examine the relationship with other related business factors during the pandemic from other countries in middle east.
• The data will more help with researches pursuing to discover the determinants of employees' work performance. It also helps in studying the family and social-professional effects of the COVID-19 crisis on employees and companies in the Saudi Arabia and Yemen. Moreover, any practical analysis related with this data will produce numerous important suggestions for other middle east countries in their fight to control social-professional effects of COVID-19 crisis on employees and businesses.

## 1 Data Description

The data set offers informative, survey-based knowledge structured as follows: respondents background (6 items), in all countries, we collect comprehensive information on contemporary job arrangements for respondents. We asked respondents to report some demographics details such as: gender, age (in years) marital status, organizational tenure (in years), education level and current jobs. Wide range of feeling about covid19 (9 items), we ask respondents to report their feeling. Though in recent weeks, governments across the world are steadily relaxing restrictions (covid-9). We want to know if still, some people also experience a wide variety of coronavirus (covid-9) event emotions, visions, perceptions, and reactions.

Feeling about job (6 items), we ask respondents to report their work details. Some professions are more exciting than others. We want to know your job's feeling. Works performance (5 items), we ask respondents to report on their emotions and how workers treat their job by making those comments. Work motivation (6 items), we ask respondents to report some statements on employee feelings over the past few weeks.

Works and family (3 items), we ask respondents to report on how workers interfere with family activities. Job satisfaction (6 items), we ask respondents to reflect on how workers think and how they spend working time. Psychological state of mind (2 items), we're asking respondents about identifying obstacles. In this questionnaire, we described challenge assessment as "a difficult situation that, while potentially stressful, you think you can overcome. These situations will help you achieve and/or inspire your work goals. On the other hand, we described hindrance situation as "something that interferes with your work and can prevent you from achieving your goals. These conditions seem like a roadblock, difficult to resolve. We want to know how employees described challenges and obstacles. Brief analysis of all abovementioned 42 items is provided as a supplementary file contains 42 figures for each item. Moreover, the questionnaire is offered as supplementary file alongside with original answers’ analysis of the respondents.

### 1.1 Descriptive statistics

To recognize and determine the situation of every concept, statistics of descriptive were utilized as a way of clarification. Table 1 displays the statistics of descriptive (standard deviation, median, mean, minimum, maximum values and degrees of freedom) for 42 items of each questionnaire's question: coronavirus (COVID-19) thought concern, employees' work performance, employees background, feeling about job, work motivation, job satisfaction, psychological state of mind and family commitment.

**Table 1:** Descriptive.

Variable	Obs	Mean	Std. Dev.	Min	Max
Q1	307	1.094463	.2929486	1	2
Q2	307	38.01303	7.008852	21	60
Q3	307	1.156352	.3726566	1	3
Q4	307	11.52932	7.900161	0	40
Q5	307	2.312704	1.016177	1	4
Q6	307	1.19544	.3971862	1	2
Q7	307	3.677524	1.264233	1	5
Q8	307	2.944625	1.512539	1	5
Q9	307	3.224756	1.454643	1	5
Q10	307	2.628664	1.236255	1	5
Q11	307	3.384365	1.336571	1	5
Q12	307	1.967427	1.257233	1	5
Q13	307	2.895765	1.379903	1	5
Q14	307	3.570033	1.399398	1	5
Q15	307	3.586319	1.258257	1	5
Q16	307	4.859935	1.784993	1	7
Q17	307	5.058632	1.67931	1	7
Q18	307	4.100977	1.966941	1	7
Q19	307	4.892508	1.827042	1	7
Q20	307	3.390879	1.941206	1	7
Q21	307	2.201954	1.059471	1	4
Q22	307	3.358306	.8216561	1	4
Q23	307	2.159609	1.065163	1	4
Q24	307	2.648208	1.038486	1	4
Q25	307	3.19544	.893465	1	4
Q26	307	4.081433	1.772119	1	7
Q27	307	3.605863	1.997801	1	7
Q28	307	3.967427	1.887068	1	7
Q29	307	5.052117	1.714191	1	7
Q30	307	4.661238	1.730889	1	7
Q31	307	3.850163	1.927703	1	7
Q32	307	3.732899	1.601264	1	6
Q37	307	2.543974	1.435038	1	6
Q39	307	2.833876	1.779079	1	6
Q33	307	2.485342	1.572521	1	6
Q34	307	3.899023	1.602561	1	6
Q35	307	2.273616	1.564534	1	6
Q36	307	3.04886	1.726634	1	6
Q38	307	2.716612	1.598963	1	6
Q40	307	1.90228	1.351673	1	6
Q41	307	3.149837	.84619	1	4
Q42	307	2.52443	1.100456	1	4

## 2 Experimental Design, Materials and Methods

The survey contains eight sections (A, B, C, D, E, F, G and H), in some sections, we used a seven- and six-point Likert scale to offer different answer options for respondents to choose. In these sections a seven- and six-point scale is the most correct and simpler to use for respondents and gives a better indication of a respondent's assessment. In this context, we used a five-point Likert scale in other sections which is relatively easier for respondents understand. We used a four-point Likert scale in some sections to get specific responses. As in this certain case of COVID-19 the opinion of respondents in these sections of the questionnaire is important so the four-point scale is the most ideal way to get it [1].

We collected a survey data on a geographically samples of people. A mixture of purposive and snowball techniques facilitated to choose the respondents via Email. After forming the questionnaire in English and Arabic with applied the suitable techniques and earlier to the formal survey, a pilot examination with 20 Academic residents from both countries was conducted to confirm the logical reliability, wording, meaning, and suitability of each question. Then we distributed 950 questionnaires through email due to the current circumstances as one time of survey data during June 1 and July 30, 2020 after Arabic world started to initiate the strict regionwide social distancing rules to cover the outbreak of COVID-19, targeting people from different sectors whom have job.

Google model was utilised as the stage for holding the questionnaire. The survey was conducted over a public online survey utilizing an appropriate sampling method. No inducements for respondents were offered, and their anonymity was assured. In this article, we offer suggestions from actual time-surveys handled in two Arabic countries the Yemen and Saudi Aribia. After data cleansing a total of 307 respondents were voluntary to participate in the survey as final sample. The original items of the Questionnaire were generated from the results of literature reviews according to previous study towards COVID-19 [2], [3], [4], [5] and the explanation about COVID-19 informed in the WHO's website [6].

The research adopted a descriptive cross-sectional online survey design to assess the dependent variable of the study, which is work output assessed by five key questions in the study questionnaire, which essentially offer us information about: finish job on time, get others to help in the job, use work time to address non-work issues and think about leaving job. Concerning feeling about COVID-19 as a moderating variable evaluated by nine questions in the study questionnaire that give us basic information about: employees' visions, expectations, emotions, thoughts and reactions.

Furthermore, independent variables calculated as follows: First variable is employee history (respondents background) measured by six questions, giving us information on: gender, age, marital status, skill / professional, job and current jobs. The second variable is job motivation assessed by six questions, giving us information about: energized, lightheaded, optimistic, emotionally stable, disturbed, downhearted, tired, stressed out and drained. The next component is family engagement (works and family) assessed by three questions, providing us with details on: personal matters, leaving the workstation for family matters and distracting responsibility from family matters.

Regarding job satisfaction variable and feeling about job each one of them measured by six and five questions respectively, giving us information about: well-satisfied, excited, negative, enjoyment and bored. Finally, psychological state of mind, variable assessed by two questions, giving us information about: feeling as a fundamental worker, daydreaming, last-minute individual and feeling frustrated. For more clarity supplementary material file labeled “Analysis of Items Answers, COVID-19″ reflects each item (Question from 1 to 42) analysis with number and the percentage of responders from whole sample.

## Ethics Statement

According to the Declaration of Helsinki the collecting data was conducted and respondents’ involvement was completely consensual, anonymous, and voluntary.

## Declaration of Competing Interest

The authors declare that they have no known opposing financial interests or personal associations that could have appeared to affect the work reported in this article.

## Appendix Supplementary materials

Image, application 1

Image, application 2

Image, application 3

Image, application 4
