# Supplementary material for: Survey data of coronavirus (COVID-19) thought concern, employees' work performance, employees background, feeling about job, work motivation, job satisfaction, psychological state of mind and family commitment in two middle east countries
Source: Data Brief. 2020 Dec 15;34:106661. doi: 10.1016/j.dib.2020.106661 (PMC7753928; doi:10.1016/j.dib.2020.106661)
Supplement: Supplementary file 2 [file mmc2.pdf]

Research on COVID-19 . أستبيان بحثي حول جائحة فيروس كورونا

السلام عليكم

في البداية أود أن أشكرك على تخصيص وقتك الثمين لإكمال الاستبيان الخاص بنا حول تأثير جائحة فيروس كورونا (COVID-19) على عملك. المعلومات التي سوف تقدمها هي لأغراض بحثية فقط. سيستغرق إكمال الاستبانة منك حوالي 5-10 دقائق. سيتم التعامل مع ردودك بسرية تامة ولن يتم الكشف عن اجاباتك مطلقًا.

تعاونك هو محل تقدير كبيرلدينا.

Assalamu Alaikum

I would like to thank you for taking your valuable time to complete our survey on how Coronavirus (COVID-19) event affects your Job. The information you are about to provide is for research purposes only. The survey will take approximately 5 -10 minutes to .finish. All of your responses will be kept strictly confidential and never disclosed your name

.Your help is greatly appreciated

If you have any questions about how to complete this questionnaire or if you have any additional comments or concerns you :would like to share regarding this survey, please feel free contact us

Mahfoudh Hussein Mgammal
alkasermr@gmail.com
Ebrahim Mohammed Al-Matari
ibrahim_matari7@yahoo.com

إذا كان لديك أي أسئلة حول كيفية ملء هذا الاستبيان أو إذا كان لديك أي ملاحظات أو مخاوف إضافية ترغب في مشاركتها معي بخصوص هذا الاستبيان ، فلا تتردد في التوصل معنا على الاميلات التالية:

alkasermr@gmail.com
ibrahim_matari7@yahoo.com

*Required

Section A: Demographics profile
معلومات عامة (A) القسم

1. Gender الجنس *

Mark only one oval.

- Female (انثى)
- Male (ذكر)

2. Marital Status *

Mark only one oval.

- Married (متزوج)
- Single (أعزب)
- Divorced or separated (مطلق)
- Other: __________

3. Age (in years) العمربالسنوات *

____

4. Employment Status نوع العمل *

Mark only one oval.

- Full-time job بدوام كلي
- Part-time بدوام جزئي

5. Level of education المستوى التعليمي *

Mark only one oval.

- PhD دكتوراة
- College or university جامعي
- Professional training متخصص
- Secondary school ثانوية
- Other: ____________________

6. Organizational tenure (in years) عدد سنوات الخدمة *

____

Section B: القسم ب

DIRECTIONS: Although in the past few weeks, governments are gradually easing (COVID-19) restrictions around the world; still, some people often to experience a wide range of feelings, dreams, thoughts and reactions about the coronavirus (COVID-19) event. For each statement below, use the following scale to indicate your agreement or disagreement with each statement using a 5-point Likert Scale ranging from 1 = Strongly disagree to 5 = Strongly agree:

على الرغم من أنه في الأسابيع القليلة الماضية، قامت الحكومات بتخفيف القيود على الناس تدريجيًا حول العالم؛ الا انه لا يزال يعاني بعض الناس في كثير من الأحيان من مجموعة واسعة من المشاعر والأحلام والأفكار وردود الفعل حول جائحة فيروس كورونا.
لكل فقرة أو عبارة من الفقرات التالية، استخدم المقياس أدناه للإشارة إلى موافقتك أو عدم موافقتك مع كل عبارة باستخدام مقياس ليكرت من 5 نقاط
تبدأ من 1 = لا أوافق بشدة إلى 5 = أوافق بشدة:

7. Despite the ease of restrictions in the past few weeks, I still feel distracted, or had to stay alert على الرغم من سهولة القيود في الأسابيع القليلة الماضية، ما زلت أشعر بالتشتت والبقاء في حالة تأهب احيانا *

Mark only one oval.

	1	2	3	4	5	
Strongly disagree لا أوافق بشدة	○	○	○	○	○	Strongly agree أوافق بشدة

8. I physically get upset or annoyed by reminders of the coronavirus (COVID-19) event أنا أنزعج حين اتذكر جائحة فيروس كورونا *

Mark only one oval.

	1	2	3	4	5	
Strongly disagree لا أوافق بشدة	○	○	○	○	○	Strongly agree أوافق بشدة

9. I still have difficulty concentrating since the recent COVID-19 pandemic crisis ما زلت أجد صعوبة في التركيز منذ أزمة جائحة فيروس كورونا الأخيرة *

Mark only one oval.

	1	2	3	4	5	
Strongly disagree لا أوافق بشدة	○	○	○	○	○	Strongly agree أوافق بشدة

10. Despite the ease of restrictions in the past few weeks, I still have felt so distant or disconnected from other people على الرغم من سهولة القيود في الأسابيع القليلة الماضية، ما زلت أشعر بأنني بعيد جدًا أو منفصل عن الآخرين *

Mark only one oval.

	1	2	3	4	5	
Strongly disagree لا أوافق بشدة	◯	◯	◯	◯	◯	Strongly agree أوافق بشدة

11. Watching coronavirus-related news and stories on traditional or social media, always make me feel as if the event is re-occurring.مشاهدة الأخبار والقصص المتعلقة بفيروس كورونا على وسائل التواصل التقليدية أو الاجتماعية تجعلني أشعر دائمًا وكأن الاحداث تتكرر مرة أخرى كل يوم *

Mark only one oval.

	1	2	3	4	5	
Strongly disagree لا أوافق بشدة	◯	◯	◯	◯	◯	Strongly agree أوافق بشدة

12. Despite the ease of restrictions in the past few weeks, I often have distressing dreams of the coronavirus (COVID-19) even. على الرغم من سهولة القيود في الأسابيع القليلة الماضية، إلا أنني غالباً لدي حلم مقلق وكئيب حول جائحة فيروس كورونا. *

Mark only one oval.

	1	2	3	4	5	
Strongly disagree لا أوافق بشدة	◯	◯	◯	◯	◯	Strongly agree أوافق بشدة

13. I am still avoiding things or going into situations which remind me about the coronavirus (COVID-19) event. مازلت أتجنب المواقف والأشياء التي تذكرني بجائحة فيروس كورونا. *

Mark only one oval.

	1	2	3	4	5	
Strongly disagree لا أوافق بشدة	◯	◯	◯	◯	◯	Strongly agree أوافق بشدة

14. I still found myself unable to remember important parts of the coronavirus (COVID-19) event مازلت أجد نفسي غير قادر على تذكر أجزاء مهمة حول جائحة فيروس كورونا *

Mark only one oval.

	1	2	3	4	5	
Strongly disagree لا أوافق بشدة	◯	◯	◯	◯	◯	Strongly agree أوافق بشدة

15. Despite the ease of restrictions in the past few weeks, I always find it difficult to fall asleep because I'm worrying about getting coronavirus (COVID-19). على الرغم من سهولة القيود في الأسابيع القليلة الماضية، إلا أنني أجد دائمًا صعوبة في النوم لأنني قلق بشأن الإصابة بالفيروس *

Mark only one oval.

	1	2	3	4	5	
Strongly disagree لا أوافق بشدة	◯	◯	◯	◯	◯	Strongly agree أوافق بشدة

Section C

DIRECTIONS: Some jobs are more interesting and satisfying than others. We want to know how you feel about your job. For each statement below, use the following scale to indicate which is most descriptive of your current job using a 7-point Likert Scale ranging from 1 = Strongly disagree to 7 = Strongly agree:

بعض الوظائف أكثر إثارة للاهتمام ومرضية من غيرها. نريد أن نعرف كيف تشعر حيال عملك.
لكل فقرة أو عبارة من الفقرات التالية، استخدم المقياس ادناة للإشارة إلى أي وصف تصف وظيفتك الحالية باستخدام مقياس ليكرت ذو 7 نقاط
يبدأ من 1 = لا أوافق بشدة إلى 7 = موافق بشدة:

16. I consider my job rather unpleasant أنا أعتبر عملي غير مرضي إلى حد ما *

Mark only one oval.

	1	2	3	4	5	6	7	
Strongly disagree لا أوافق بشدة	◯	◯	◯	◯	◯	◯	◯	Strongly agree أوافق بشدة

17. I find real enjoyment in my work أجد متعة حقيقية في عملي *

Mark only one oval.

	1	2	3	4	5	6	7	
Strongly disagree لا أوافق بشدة	◯	◯	◯	◯	◯	◯	◯	Strongly agree أوافق بشدة

18. Each day of work seems like it will never end كل يوم عمل يبدو لي أن ليس له نهاية *

Mark only one oval.

	1	2	3	4	5	6	7	
Strongly disagree لا أوافق بشدة	◯	◯	◯	◯	◯	◯	◯	Strongly agree أوافق بشدة

19. I feel fairly well satisfied with my present job أشعر بالرضا إلى حد ما عن عملي الحالي *

Mark only one oval.

	1	2	3	4	5	6	7	
Strongly disagree لا أوافق بشدة	◯	◯	◯	◯	◯	◯	◯	Strongly agree أوافق بشدة

20. Most days I am enthusiastic about my work أنا متحمس في معظم الأيام لعملي *

Mark only one oval.

	1	2	3	4	5	6	7	
Strongly disagree لا أوافق بشدة	◯	◯	◯	◯	◯	◯	◯	Strongly agree أوافق بشدة

Section D

DIRECTIONS: Please use a scale with 1 = Strongly disagree to 4 = Strongly agree to indicate each statement that is most applicable to you.

لكل فقرة أو عبارة من الفقرات التالية، استخدم المقياس ادناة للإشارة إلى موافقتك أو عدم موافقتك مع كل عبارة باستخدام مقياس ليكرت من 4 نقاط
تبدأ من 1 = لا أوافق بشدة إلى 4 = أوافق بشدة:

21. I always leave my tasks to the last minute. أترك دائما مهماتي حتى اللحظة الأخيرة *

Mark only one oval.

	1	2	3	4	
Strongly disagree لا أوافق بشدة	◯	◯	◯	◯	Strongly agree أوافق بشدة

22. Sometimes, I feel disappointed with my performance at work, because I know I could have done better في بعض الأحيان ، أشعر بخيبة أمل من أدائي في العمل، لأنني أعلم أنه كان بوسعي تحقيق أداء أفضل *

Mark only one oval.

	1	2	3	4	
Strongly disagree لا أوافق بشدة	◯	◯	◯	◯	Strongly agree أوافق بشدة

23. I consider myself a fundamental worker to the organization I work for, due to the high quality of my performance أنا أعتبر نفسي عاملاً أساسيًا في المؤسسة التي أعمل فيها، نظرًا للجودة العالية لأدائي *

Mark only one oval.

	1	2	3	4	
Strongly disagree لا أوافق بشدة	◯	◯	◯	◯	Strongly agree أوافق بشدة

24. When I have a deadline to perform a certain task, I always finish it on time. عندما يكون لدي موعد نهائي لأداء مهمة معينة، أقوم دائمًا بإنهائه في الوقت المحدد *

Mark only one oval.

	1	2	3	4	
Strongly disagree لا أوافق بشدة	◯	◯	◯	◯	Strongly agree أوافق بشدة

25. It is not always easy for me to perform tasks on time. ليس من السهل علي دائمًا القيام بالمهام في الوقت المحدد *

Mark only one oval.

	1	2	3	4	
Strongly disagree لا أوافق بشدة	◯	◯	◯	◯	Strongly agree أوافق بشدة

Section E

DIRECTIONS: Select one response for each of the following statement to indicate your agreement or disagreement using a 7-point Likert Scale ranging from 1 = Strongly disagree to 7 = Strongly agree.

حدد إجابة واحدة لكل بيان من العبارات التالية للإشارة إلى موافقتك أو عدم موافقتك باستخدام مقياس ليكرت ذو 7 نقاط
يبدأ من 1 = لا أوافق بشدة إلى 7 = أوافق بشدة

26. I was emotionally stable and sure of myself during the past few weeks. كنت مستقرا عاطفيا وواثق من نفسي خلال الأسابيع القليلة الماضية. *

Mark only one oval.

	1	2	3	4	5	6	7	
Strongly disagree لا أوافق بشدة	◯	◯	◯	◯	◯	◯	◯	Strongly agree أوافق بشدة

27. I felt cheerful, lighthearted during the past few weeks. شعرت بالبهجة والراحة خلال الأسابيع القليلة الماضية *

Mark only one oval.

	1	2	3	4	5	6	7	
Strongly disagree لا أوافق بشدة	◯	◯	◯	◯	◯	◯	◯	Strongly agree أوافق بشدة

28. I felt tired, worn out, used up, or exhausted during the past few weeks. شعرت بالتعب والإرهاق وكنت منهك خلال الأسابيع القليلة الماضية *

Mark only one oval.

	1	2	3	4	5	6	7	
Strongly disagree	◯	◯	◯	◯	◯	◯	◯	Strongly agree أوافق بشدة

29. I felt bothered during the past few weeks شعرت بالانزعاج خلال الأسابيع القليلة الماضية *

Mark only one oval.

	1	2	3	4	5	6	7	
Strongly disagree لا أوافق بشدة	◯	◯	◯	◯	◯	◯	◯	Strongly agree أوافق بشدة

30. I felt downhearted and blue during the past few weeks شعرت بالأسى والكنابة خلال الأسابيع القليلة الماضية *

Mark only one oval.

	1	2	3	4	5	6	7	
Strongly disagree لا أوافق بشدة	◯	◯	◯	◯	◯	◯	◯	Strongly agree أوافق بشدة

31. I felt much energy, pep, or vitality during the past few weeks شعرت بالكثير من الطاقة والحيوية والنشاط خلال الأسابيع القليلة الماضية *

Mark only one oval.

	1	2	3	4	5	6	7	
Strongly disagree لا أوافق بشدة	◯	◯	◯	◯	◯	◯	◯	Strongly agree أوافق بشدة

Section F

DIRECTIONS: We want to know how your work responsibilities interfere with family activities. For each statement below, use the following scale to indicate which is most descriptive of your current job using a 4-point Likert Scale ranging from 1 = Strongly disagree to 4 = Strongly agree:

نريد أن نعرف كيف تتداخل مسؤوليات عملك مع الأنشطة العائلية لذا لكل فقرة أو عبارة من الفقرات التالية، استخدم المقياس ادناة للإشارة إلى موافقتك أو عدم موافقتك مع كل عبارة باستخدام مقياس ليكرت من 4 نقاط
تبدأ من 1 = لا أوافق بشدة إلى 4 = أوافق بشدة:

32. The time I spend on family responsibilities predominantly interferes with my work responsibilities. غالبًا ما يتعارض الوقت الذي أمضيه في المسؤوليات العائلية مع مسؤوليات عملي. *

Mark only one oval.

	1	2	3	4	
Strongly disagree لا أوافق بشدة	◯	◯	◯	◯	Strongly agree أوافق بشدة

Section G

DIRECTIONS: Keeping in mind that all your responses will be kept anonymous, use 6-point Likert Scale ranging from 1 = Strongly disagree to 6 = Strongly agree to indicate in the past few weeks how often have you the following:

مع الأخذ في الاعتبار أن جميع ردودك ستبقى سرية ومجهولة المصدر، استخدم مقياس ليكرت من 6 نقاط تبدأ من 1 = لا أوافق بشدة إلى 6 = أوافق بشدة للإشارة إلى عدد المرات التي قمت في الأسابيع القليلة الماضية بما يلي:

33. Spent work time on personal matters قضاء وقت العمل في الأمور الشخصية *

Mark only one oval.

	1	2	3	4	5	6	
Strongly disagree لا أوافق بشدة	◯	◯	◯	◯	◯	◯	Strongly agree أوافق بشدة

34. Put less effort into job than should have بذل جهد أقل في العمل مما كان ينبغي *

Mark only one oval.

	1	2	3	4	5	6	
Strongly disagree لا أوافق بشدة	◯	◯	◯	◯	◯	◯	Strongly agree أوافق بشدة

35. Let others do your work تترك الآخرين يقومون بعملك *

Mark only one oval.

	1	2	3	4	5	6	
Strongly disagree لا أوافق بشدة	◯	◯	◯	◯	◯	◯	Strongly agree أوافق بشدة

36. Daydreaming احلام اليقظة *

Mark only one oval.

	1	2	3	4	5	6	
Strongly disagree لا أوافق بشدة	◯	◯	◯	◯	◯	◯	Strongly agree أوافق بشدة

37. Thoughts of leaving current job التفكير في ترك الوظيفة الحالية *

Mark only one oval.

	1	2	3	4	5	6	
Strongly disagree لا أوافق بشدة	◯	◯	◯	◯	◯	◯	Strongly agree أوافق بشدة

38. Discuss with coworkers about non-work issues ناقشت مع زملاء العمل قضايا لا تتعلق بالعمل *

Mark only one oval.

	1	2	3	4	5	6	
Strongly disagree لا أوافق بشدة	◯	◯	◯	◯	◯	◯	Strongly agree أوافق بشدة

39. Thoughts of being absent التفكير في الغياب عن العمل *

Mark only one oval.

	1	2	3	4	5	6	
Strongly disagree لا أوافق بشدة	◯	◯	◯	◯	◯	◯	Strongly agree أوافق بشدة

40. Left work station for unnecessary reasons ترك مكان العمل لأسباب غير ضرورية *

Mark only one oval.

	1	2	3	4	5	6	
Strongly disagree لا أوافق بشدة	◯	◯	◯	◯	◯	◯	Strongly agree أوافق بشدة

Section H

DIRECTIONS: In this questionnaire, I defined challenge appraisal "as a challenging circumstance that although potentially stressful that you think you can overcome. These circumstances can help you meet your work goals and/or be motivating". On the other hand, I defined hindrance circumstance "as something that interferes with your work and can stand in the way of you being able to achieve your goals. These circumstances seem almost as a road block, impossible to overcome".

من ناحية أخرى، قمت بتعريف العوائق كتالي:
"كشيء يتداخل مع عملك ويمكن أن يقف في طريق قدرتك على تحقيق أهدافك. وتبدو هذه الظروف وكأنها حاجز طريق، من المستحيل التغلب عليها".

في هذا الاستبيان، قد عرفت التحدي كالتالي:
"كظرف صعب من المحتمل أن يكون مرهقًا وتعتقد أنه يمكنك التغلب عليه. ويمكن أن تساعدك هذه الظروف على تحقيق أهداف عملك وأن تكون محفزًا".

41. Coronavirus (COVID-19) event is a hindrance circumstance that completely interferes with my work and making difficult to achieve my work goals. Therefore, COVID-19 event appear to be a road block and impossible to overcome. جائحة كرونا عائق يتداخل مع عملك ويمكن أن يقف في طريق قدرتك على تحقيق أهدافك. وتبدو هذه الظروف وكأنها حاجز طريق، من المستحيل التغلب عليها".

Mark only one oval.

	1	2	3	4	
Strongly disagree لا أوافق بشدة	◯	◯	◯	◯	Strongly agree أوافق بشدة

42. Based on these definitions, use the following scale to indicate your agreement or disagreement with each statement using a 4-point Likert Scale ranging from 1 = Strongly disagree to 4 = Strongly agree: Coronavirus (COVID-19) event is a challenging circumstance that although potentially stressful, I think I could overcome it. This is because COVID-19 event is motivating circumstance that can help me meet my work goals. بناءً على هذه التعريفات السابقة، استخدم المقياس ادناه للإشارة إلى موافقتك أو عدم موافقتك مع كل عبارة باستخدام مقياس ليكرت من 4 نقاط يبدأ من 1 = لا أوافق بشدة إلى 4 = أوافق بشدة: وتعد جائحة كرونا ظرفًا صعبًا ومرهقًا، وتعتقد أنه يمكنك التغلب عليه. هذا لأنه حدث محفز للظروف التي يمكن أن تساعدنك على تحقيق أهداف عملك

Mark only one oval.

	1	2	3	4	
Strongly disagree لا أوافق بشدة	◯	◯	◯	◯	Strongly agree أوافق بشدة

This content is neither created nor endorsed by Google.

Google Forms
