# Supplementary material for: Survey data of coronavirus (COVID-19) thought concern, employees' work performance, employees background, feeling about job, work motivation, job satisfaction, psychological state of mind and family commitment in two middle east countries
Source: Data Brief. 2020 Dec 15;34:106661. doi: 10.1016/j.dib.2020.106661 (PMC7753928; doi:10.1016/j.dib.2020.106661)
Supplement: Supplementary file 3 [file mmc3.pdf]

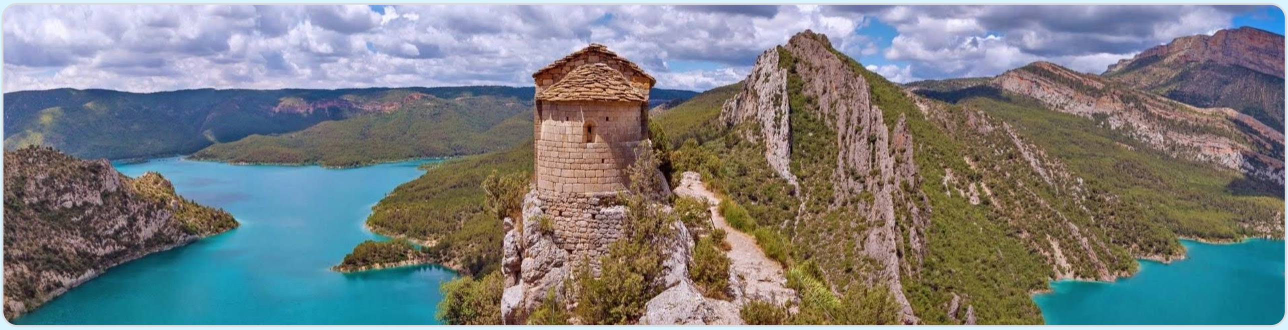

Research on COVID-19 . أستبيان بحثي حول جائحة فيروس كورونا

307 responses

Publish analytics

Section A: Demographics profile

Gender الجنس

307 responses

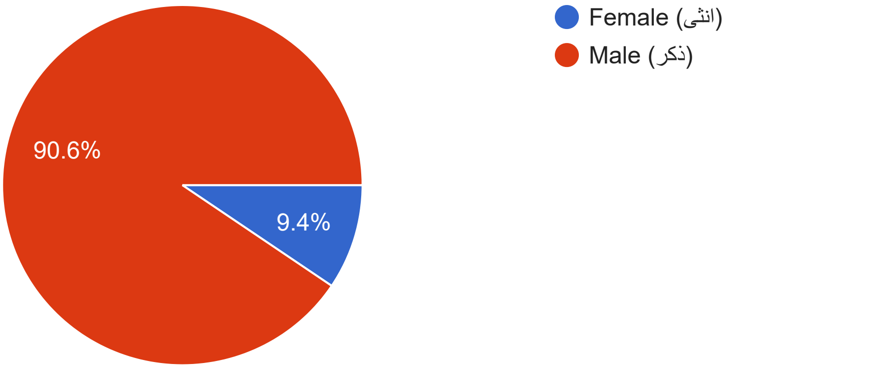

Marital Status

307 responses

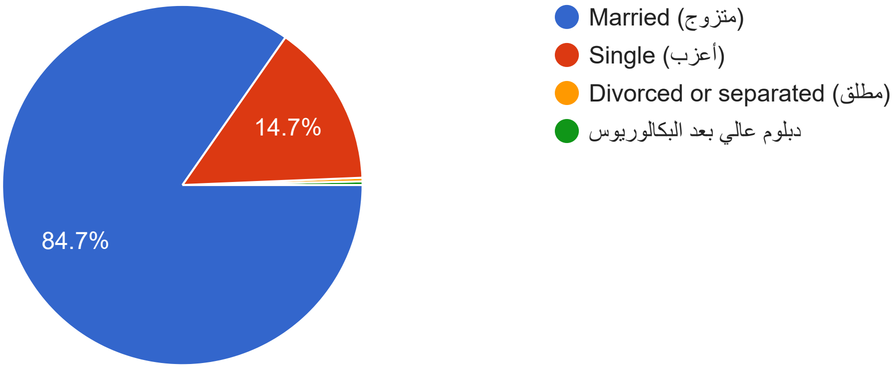

Age (in years) العمربالسنوات

307 responses

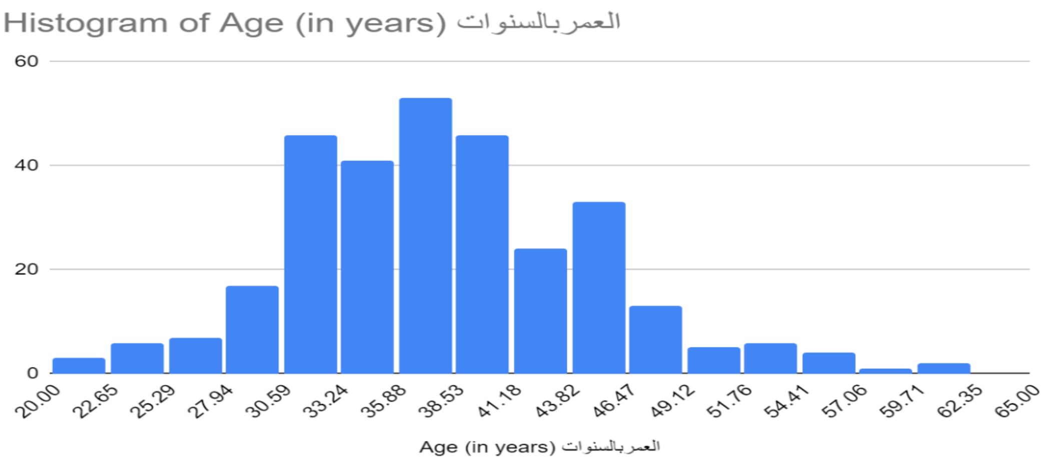

Employment Status نوع العمل

307 responses

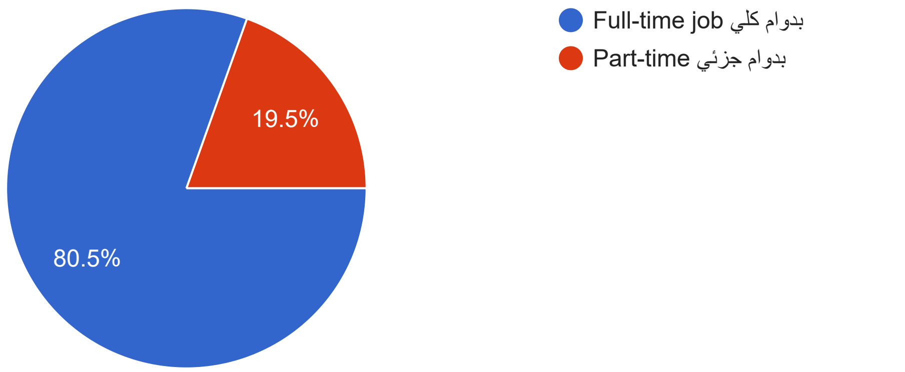

Level of education المستوى التعليمي

307 responses

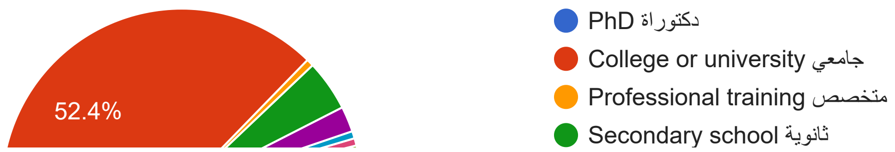

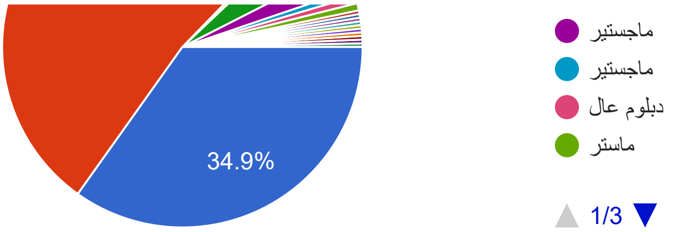

Organizational tenure (in years) عدد سنوات الخدمة

307 responses

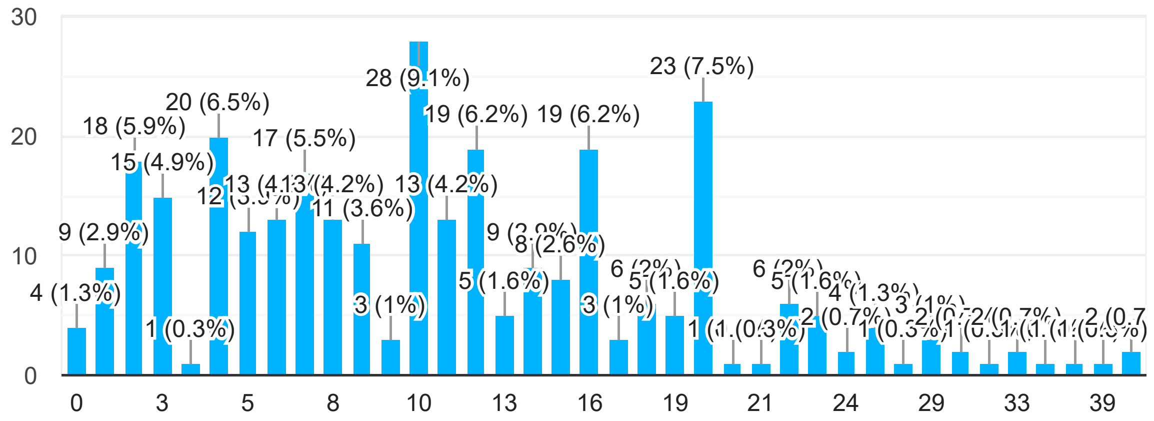

Section B القسم: ب

Despite the ease of restrictions in the past few weeks, I still feel distracted, or had to stay alert على الرغم من سهولة القيود في الأسابيع القليلة الماضية، ما زلت أشعر بالتشتت والبقاء في حالة تأهب احياناً

307 responses

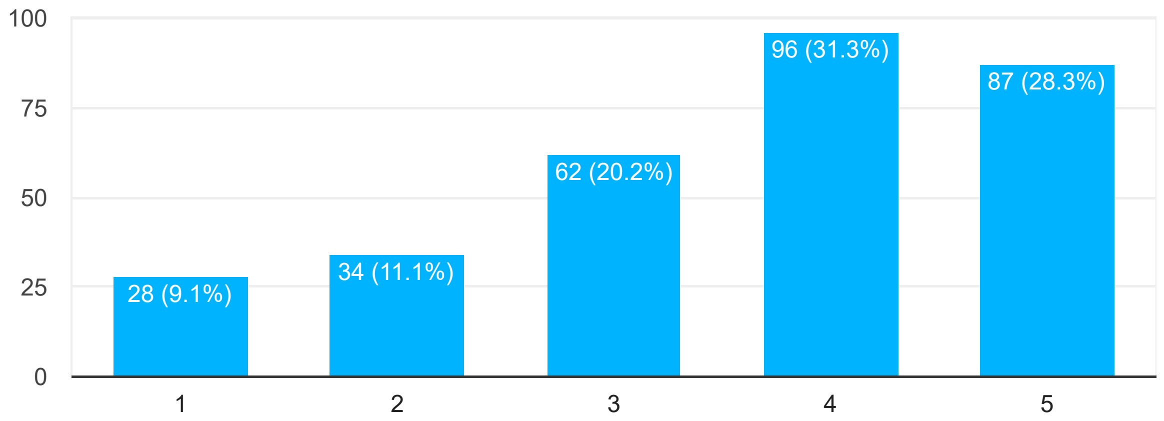

I physically get upset or annoyed by reminders of the coronavirus (COVID-19) event أنا أنزعج حين اتذكر جائحة فيروس كورونا

307 responses

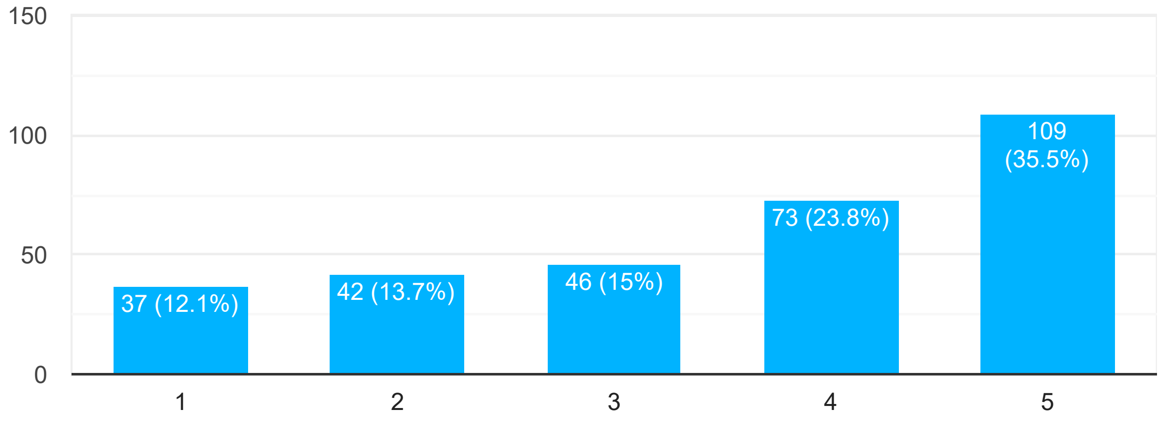

I still have difficulty concentrating since the recent COVID-19 pandemic crisis ما زلت أجد صعوبة في التركيز منذ أزمة جائحة فيروس كورونا الأخيرة

307 responses

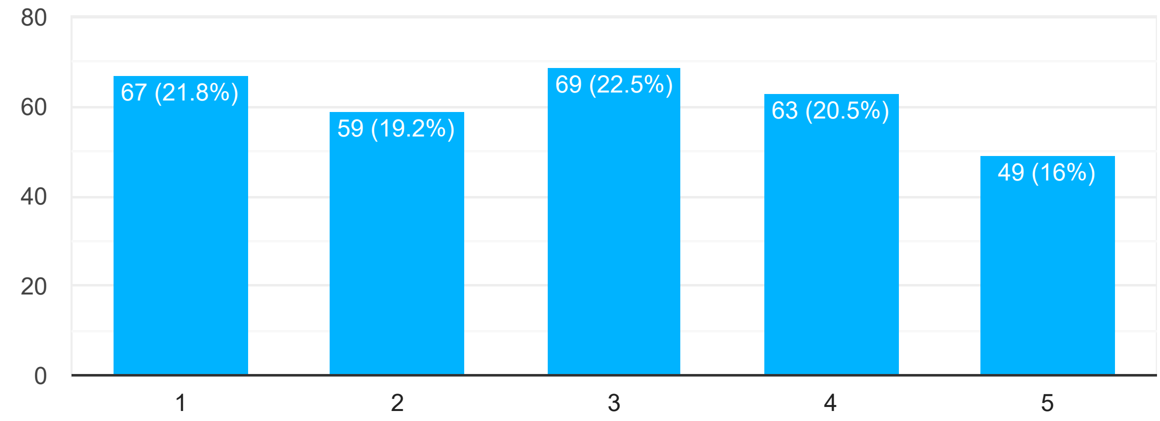

Despite the ease of restrictions in the past few weeks, I still have felt so distant or disconnected from other people على الرغم من سهولة القيود في الأسابيع القليلة الماضية، ما زلت أشعر بأنني بعيد جدًا أو منفصل عن الآخرين

307 responses

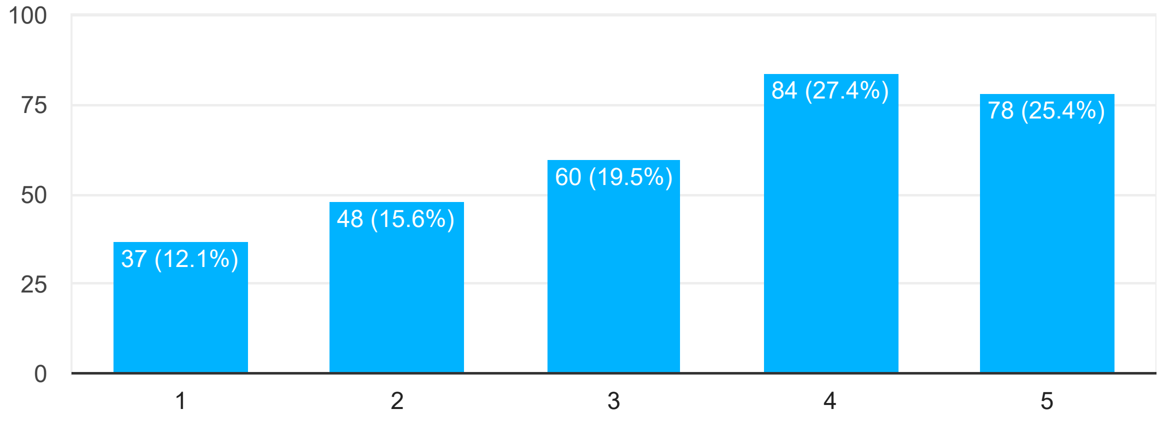

Watching coronavirus-related news and stories on traditional or social media, always make me feel as if the event is re-occurring.مشاهدة الأخبار والقصص المتعلقة بفيروس كورونا على وسائل التواصل التقليدية أو الاجتماعية تجعلني أشعر دائمًا وكأن الاحداث تتكرر مرة أخرى كل يوم

307 responses

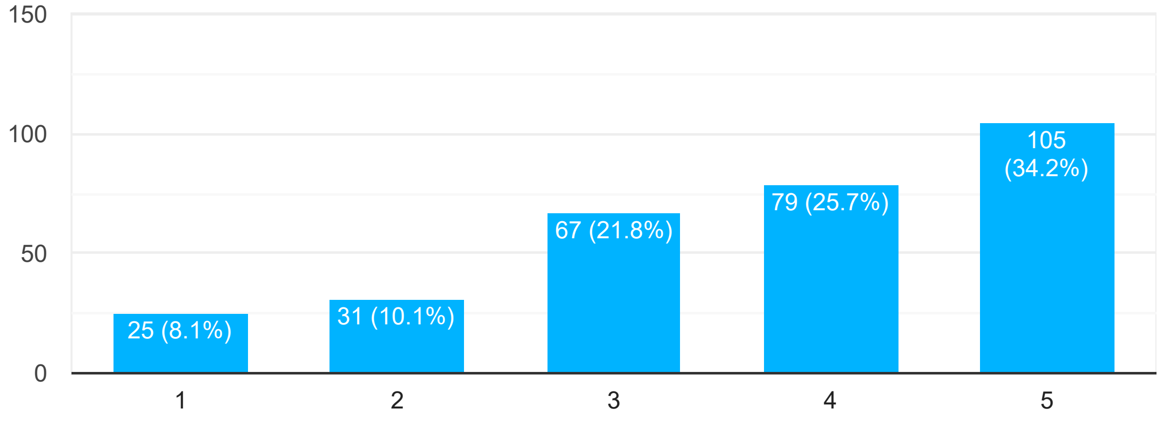

Despite the ease of restrictions in the past few weeks, I often have distressing dreams of the coronavirus (COVID-19) even. على الرغم من سهولة القيود في الأسابيع القليلة الماضية، إلا أنني غالباً لدي حلم مقلق وكئيب حول جائحة فيروس كورونا.

307 responses

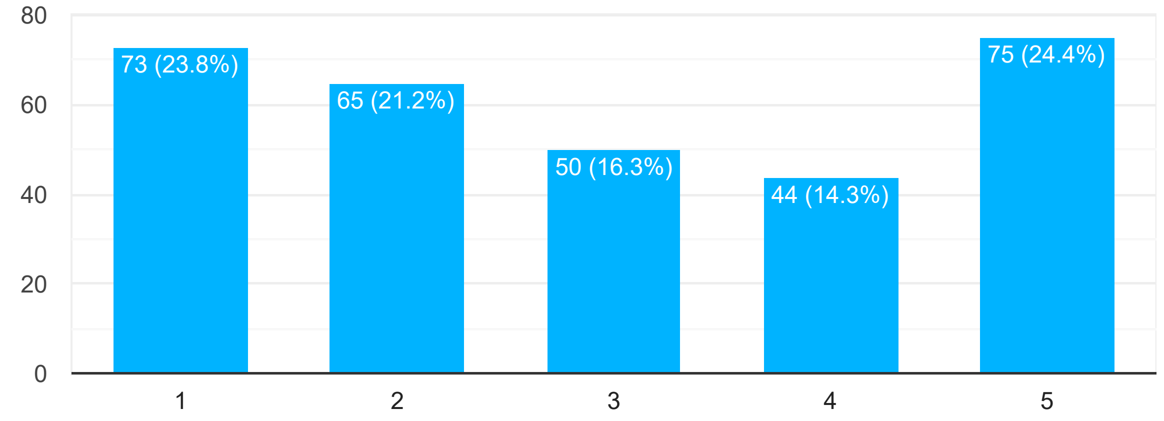

I am still avoiding things or going into situations which remind me about the coronavirus (COVID-19) event. مازلت أتجنب المواقف والأشياء التي تذكرني بجائحة فيروس كورونا.

307 responses

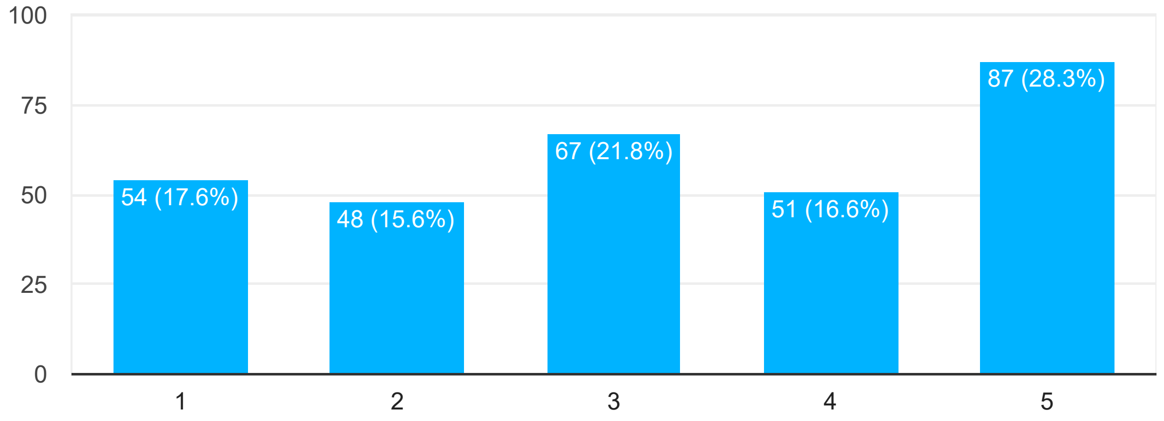

I still found myself unable to remember important parts of the coronavirus (COVID-19) event مازلت أجد نفسي غير قادر على تذكر أجزاء مهمة حول جائحة فيروس كورونا

307 responses

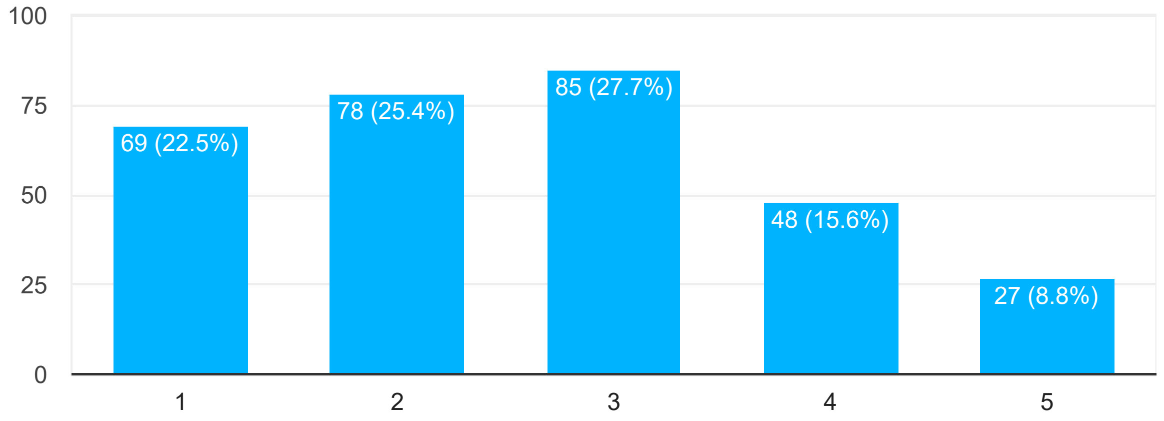

Despite the ease of restrictions in the past few weeks, I always find it difficult to fall asleep because I'm worrying about getting coronavirus (COVID-19). على الرغم من سهولة القيود في الأسابيع القليلة الماضية، إلا أنني أجد دائمًا صعوبة في النوم لأنني قلق بشأن الإصابة بالفيروس

307 responses

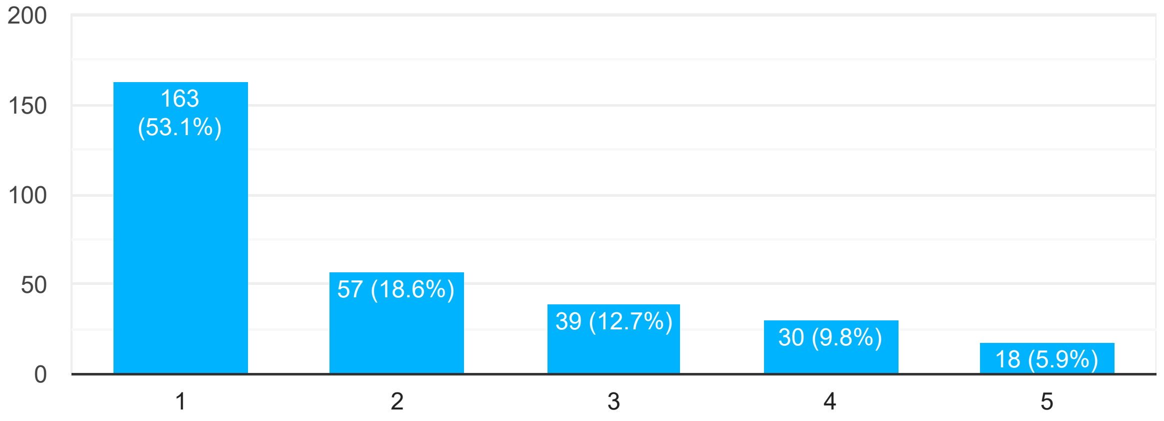

Section C

I consider my job rather unpleasant أنا أعتبر عملي غير مرضي إلى حد ما

307 responses

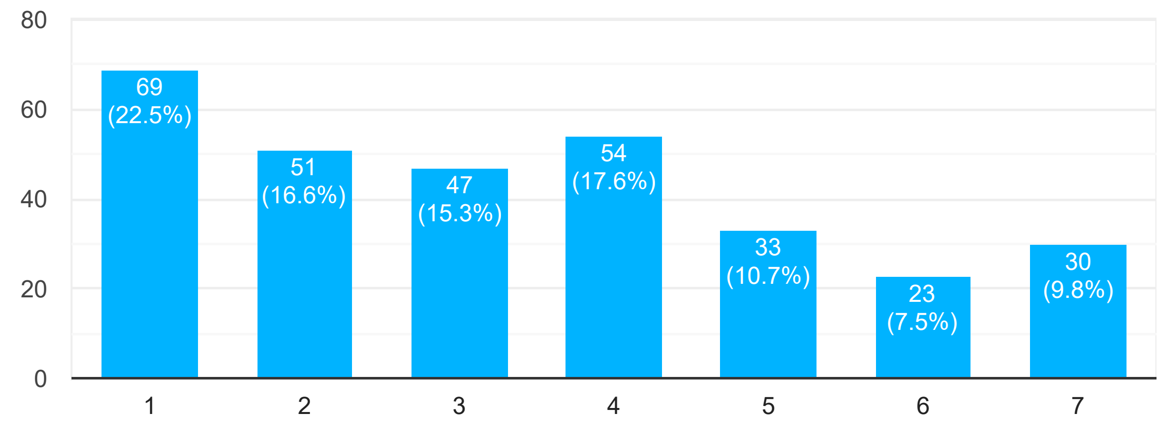

I find real enjoyment in my work أجد متعة حقيقية في عملي

307 responses

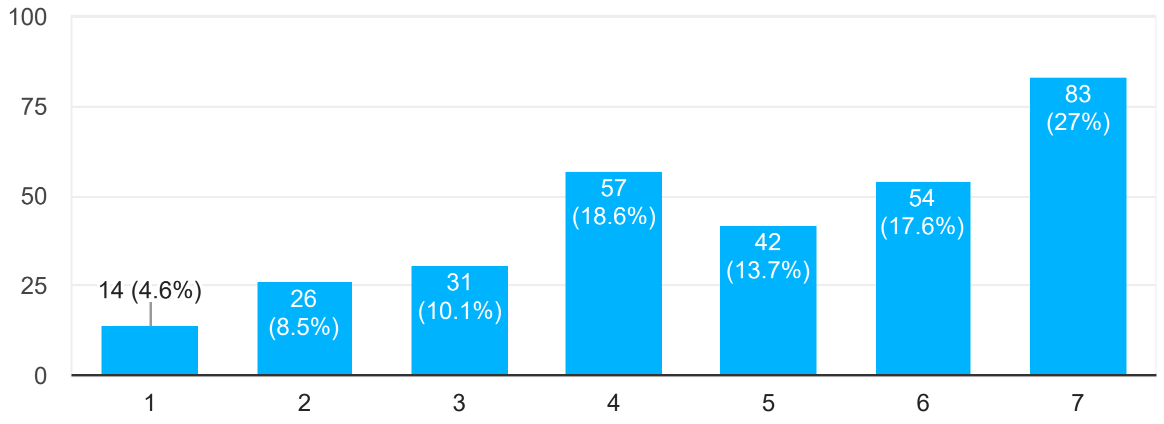

Each day of work seems like it will never end كل يوم عمل يبدو لي أن ليس له نهاية

307 responses

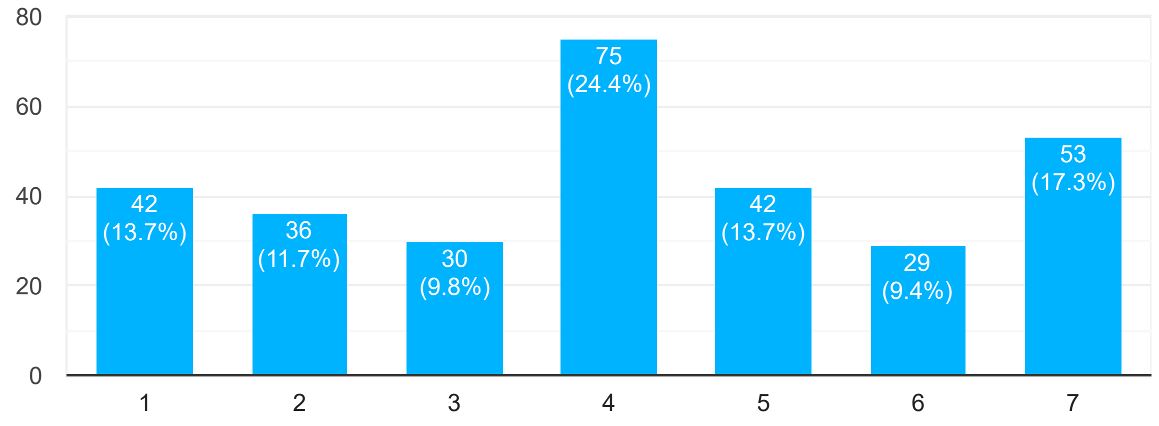

I feel fairly well satisfied with my present job أشعر بالرضا إلى حد ما عن عملي الحالي

307 responses

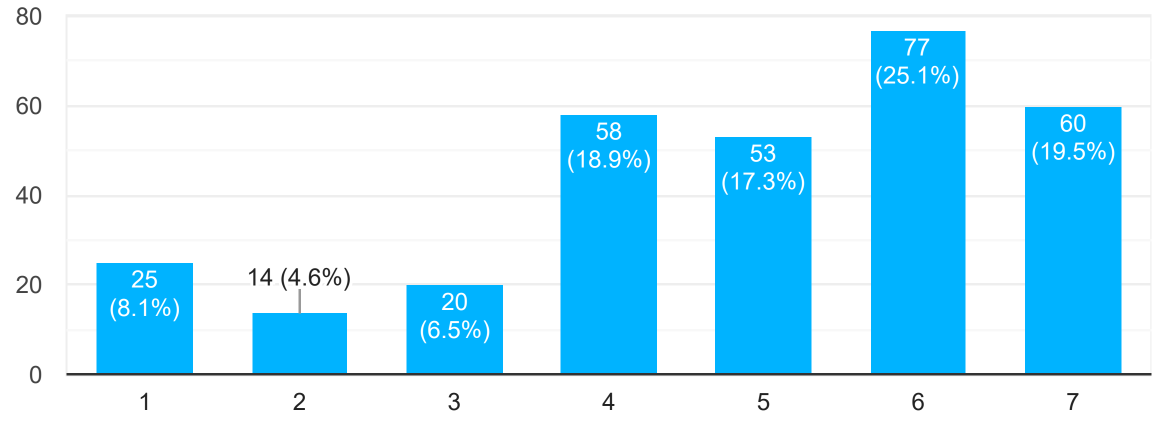

Most days I am enthusiastic about my work أنا متحمس في معظم الأيام لعملي

307 responses

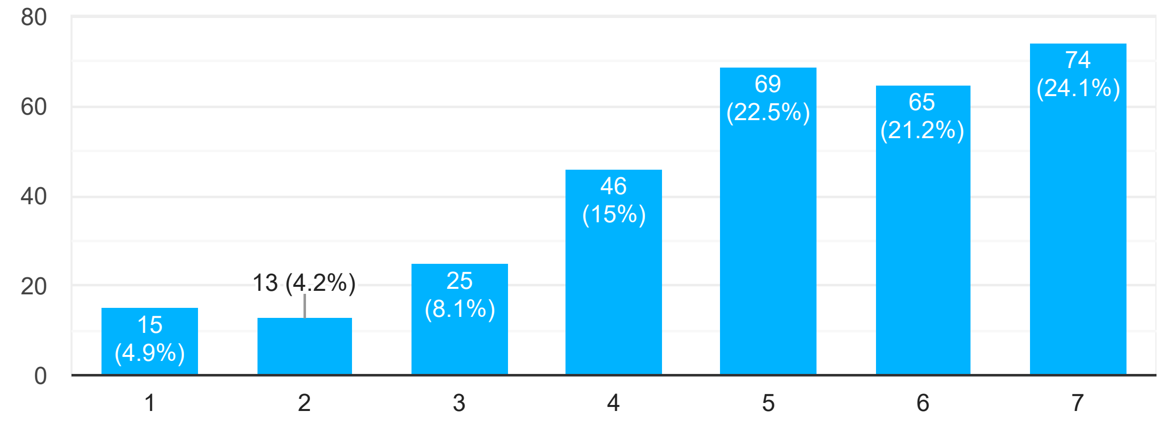

Section D

I always leave my tasks to the last minute. أترك دائما مهماتي حتى اللحظة الأخيرة

307 responses

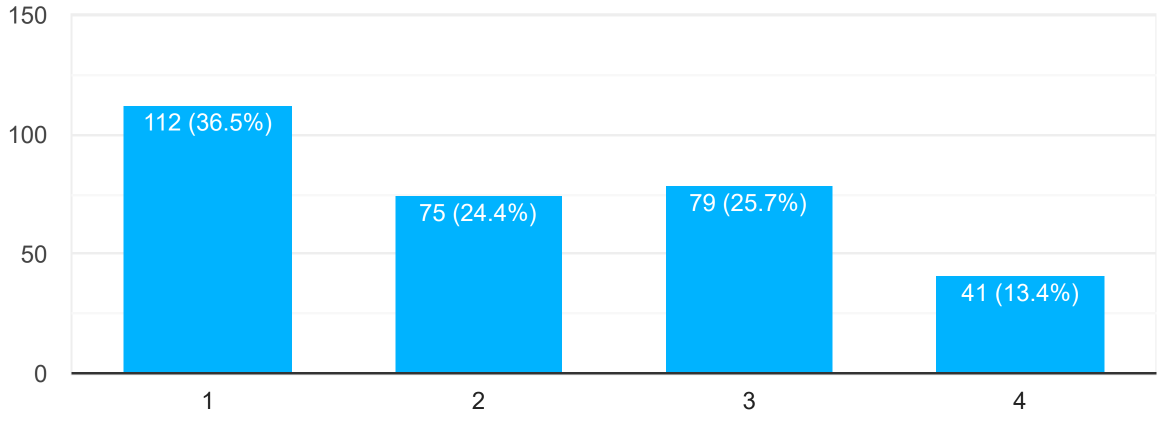

Sometimes, I feel disappointed with my performance at work, because I know I could have done better في بعض الأحيان ، أشعر بخيبة أمل من أدائي في العمل، لأنني أعلم أنه كان بوسعي تحقيق أداء أفضل

307 responses

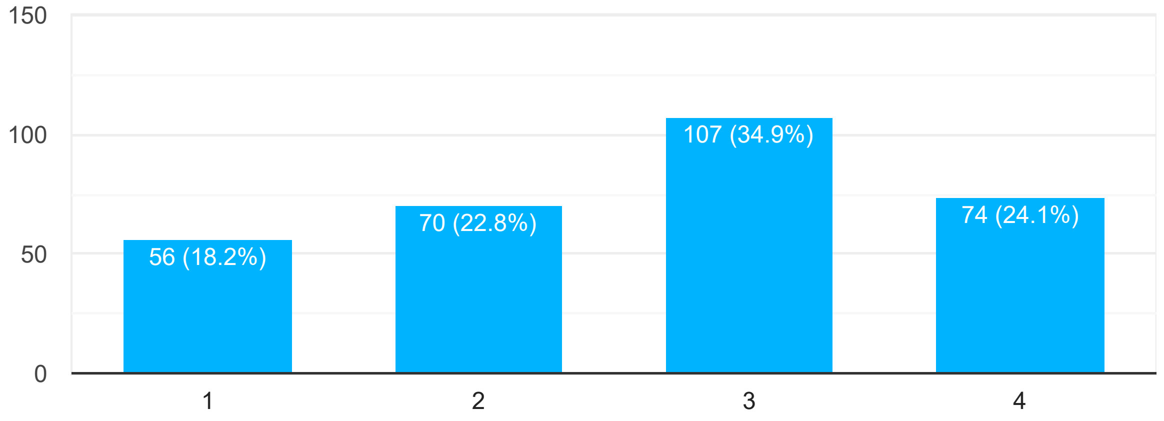

I consider myself a fundamental worker to the organization I work for, due to the high quality of my performance أنا أعتبر نفسي عاملاً أساسيًا في المؤسسة التي أعمل فيها، نظرًا للجودة العالية لأدائي

307 responses

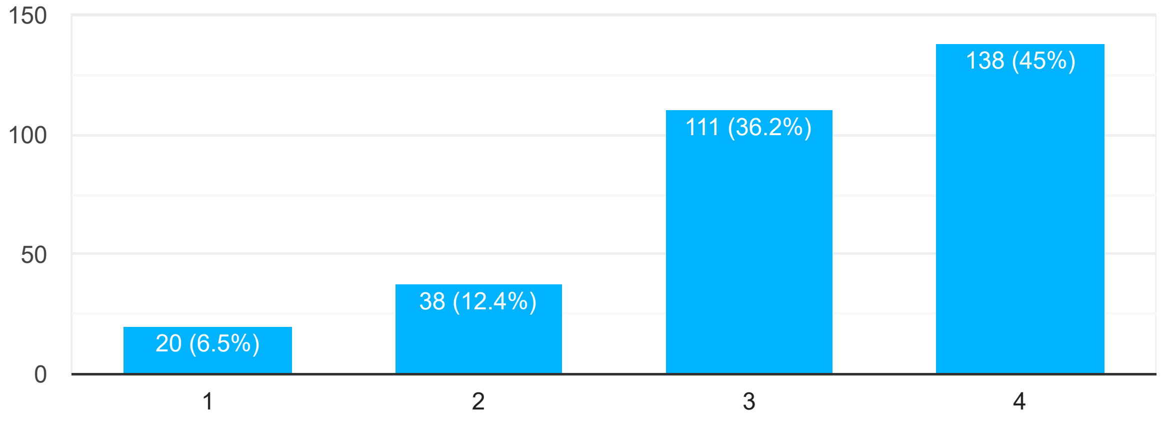

When I have a deadline to perform a certain task, I always finish it on time.
عندما يكون لدي موعد نهائي لأداء مهمة معينة، أقوم دائمًا بإنهائه في الوقت المحدد

307 responses

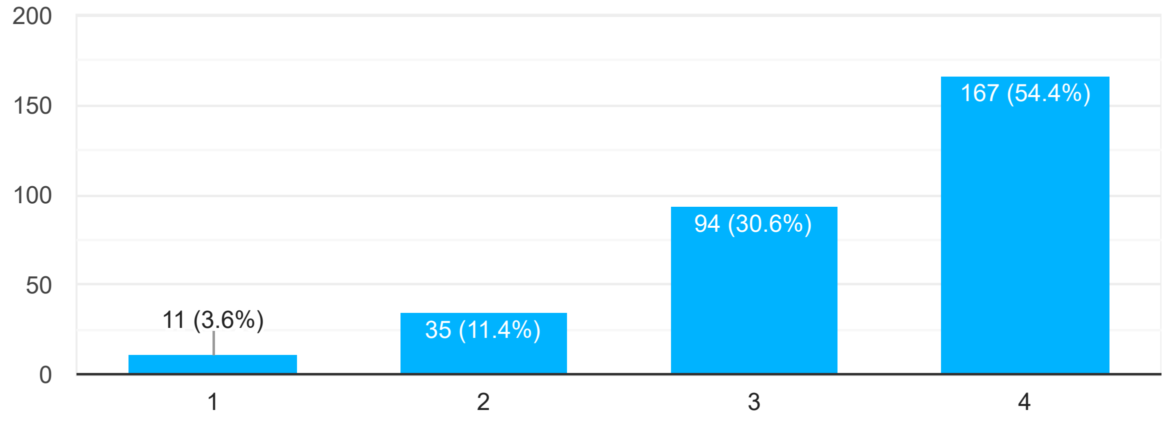

It is not always easy for me to perform tasks on time. ليس من السهل علي دائمًا القيام بالمهام في الوقت المحدد

307 responses

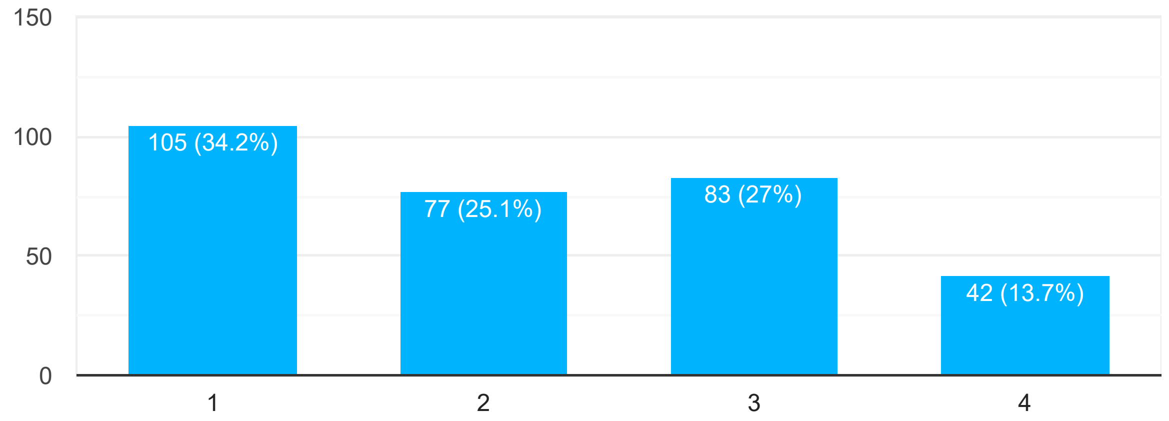

Section E

I was emotionally stable and sure of myself during the past few weeks. كنت مستقرا عاطفيا وواثق من نفسي خلال الأسابيع القليلة الماضية.

307 responses

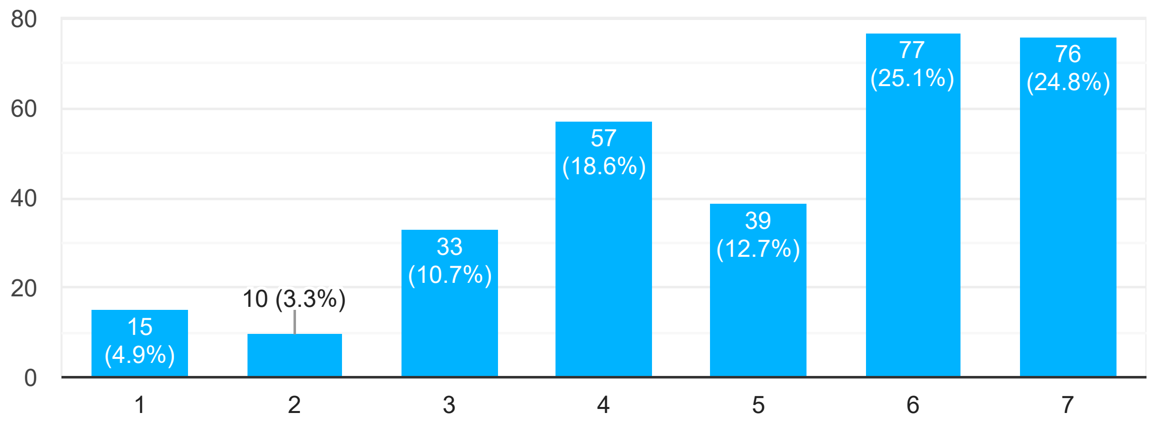

I felt cheerful, lighthearted during the past few weeks. شعرت بالبهجة والراحة خلال الأسابيع القليلة الماضية

307 responses

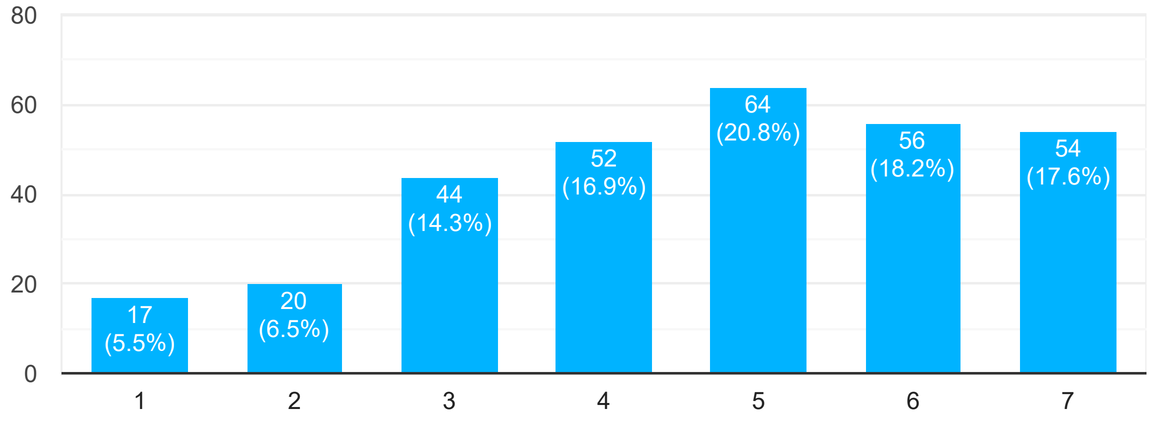

I felt tired, worn out, used up, or exhausted during the past few weeks.
شعرت بالتعب والإرهاق وكنت منهك خلال الأسابيع القليلة الماضية

307 responses

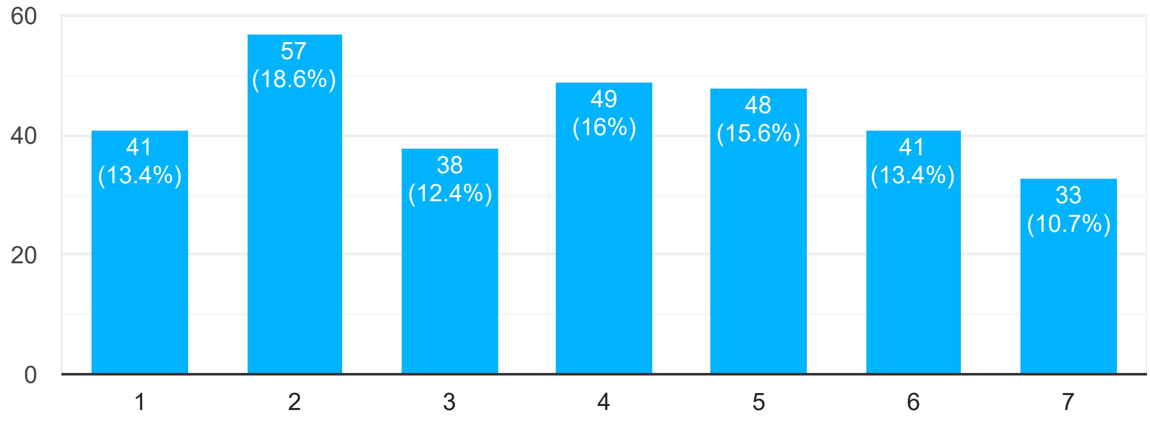

I felt bothered during the past few weeks شعرت بالانزعاج خلال الأسابيع القليلة الماضية

307 responses

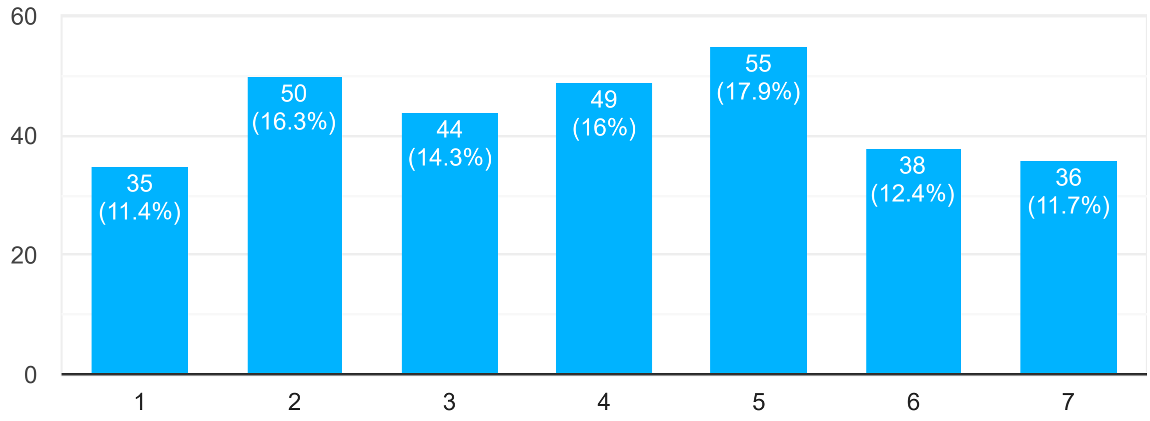

I felt downhearted and blue during the past few weeks شعرت بالأسى والكئابة خلال الأسابيع القليلة الماضية

307 responses

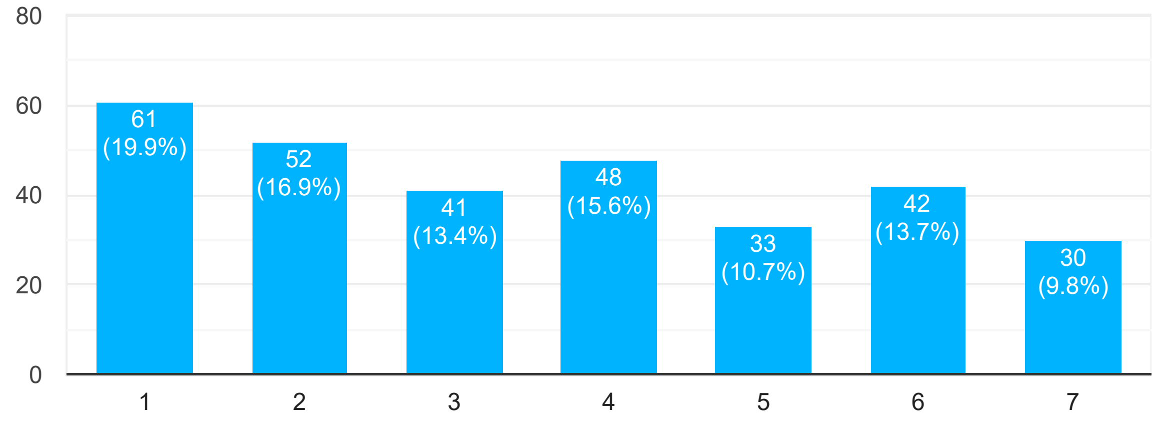

I felt much energy, pep, or vitality during the past few weeks شعرت بالكثير من الطاقة والحيوية والنشاط خلال الأسابيع القليلة الماضية

307 responses

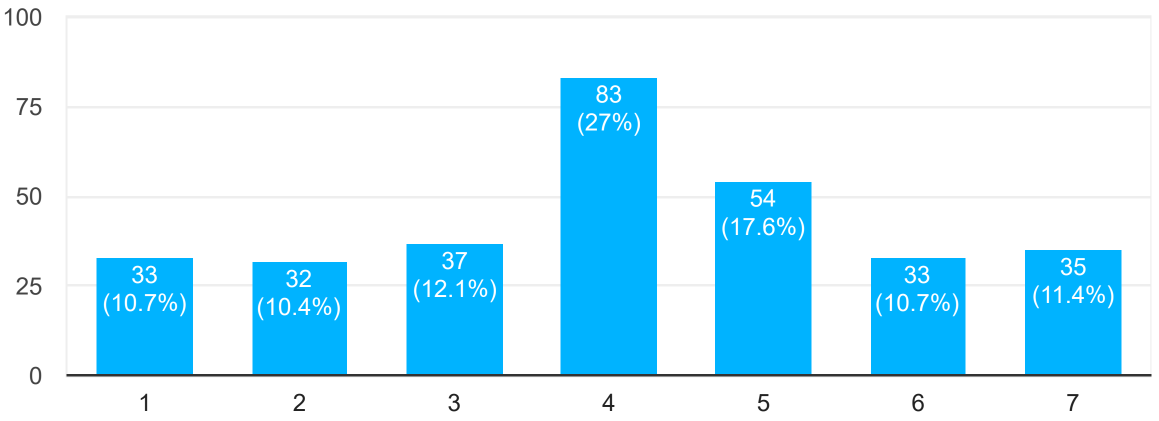

Section F

The time I spend on family responsibilities predominantly interferes with my work responsibilities. غالبًا ما يتعارض الوقت الذي أمضيه في المسؤوليات العائلية مع مسؤوليات عملي.

307 responses

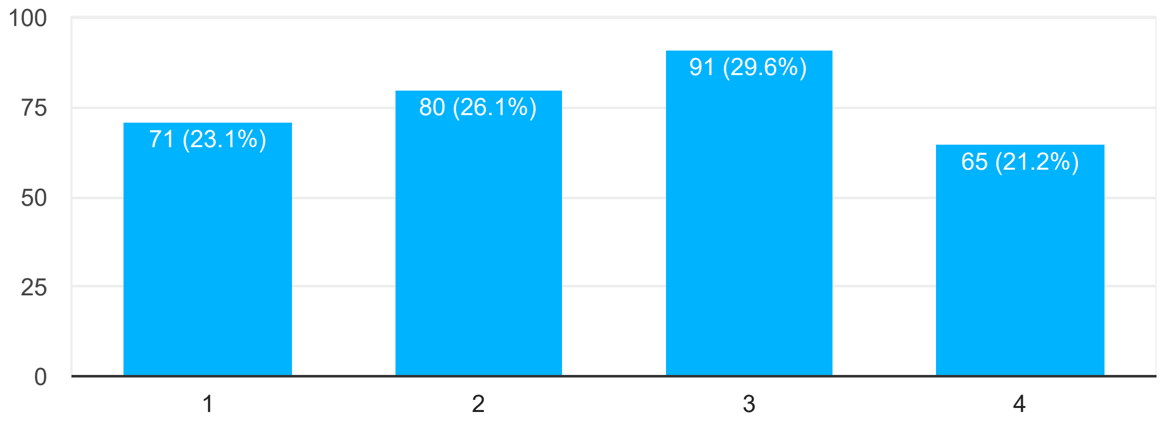

Section G

Spent work time on personal matters قضاء وقت العمل في الأمور الشخصية

307 responses

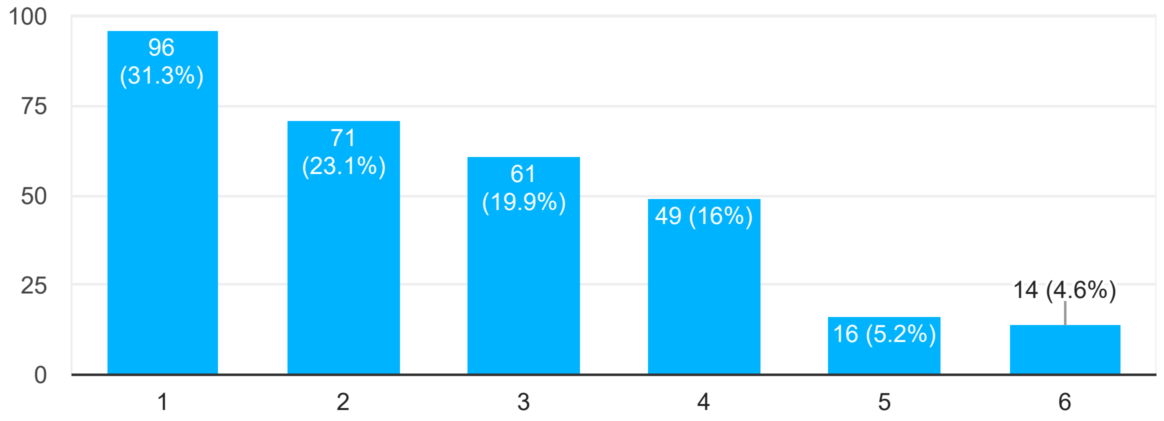

Put less effort into job than should have بذل جهد أقل في العمل مما كان ينبغي

307 responses

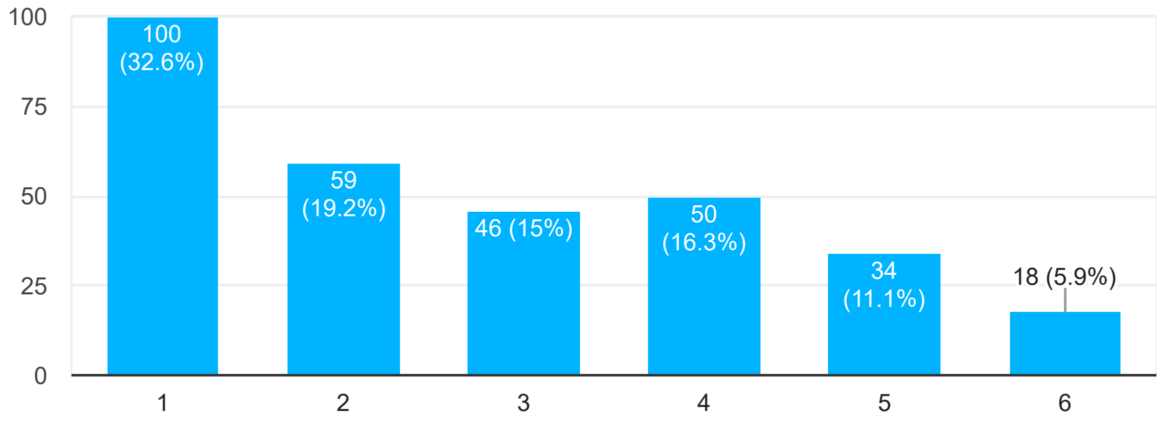

Let others do your work تترك الآخرين يقومون بعملك

307 responses

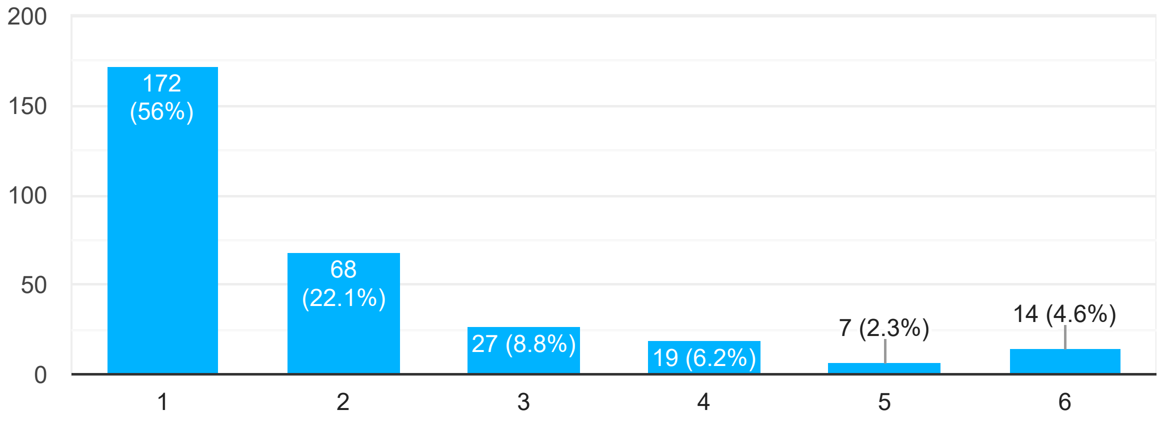

Daydreaming احلام اليقظة

307 responses

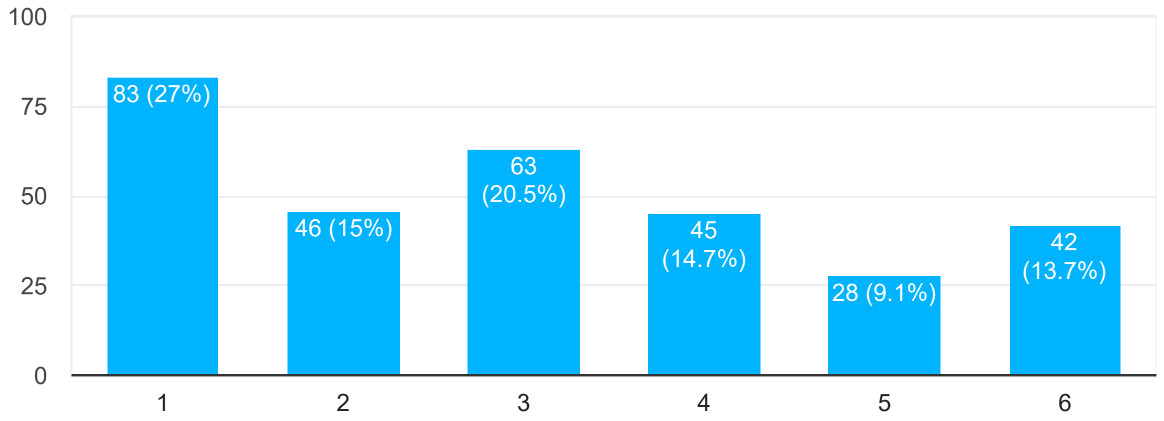

Thoughts of leaving current job التفكير في ترك الوظيفة الحالية

307 responses

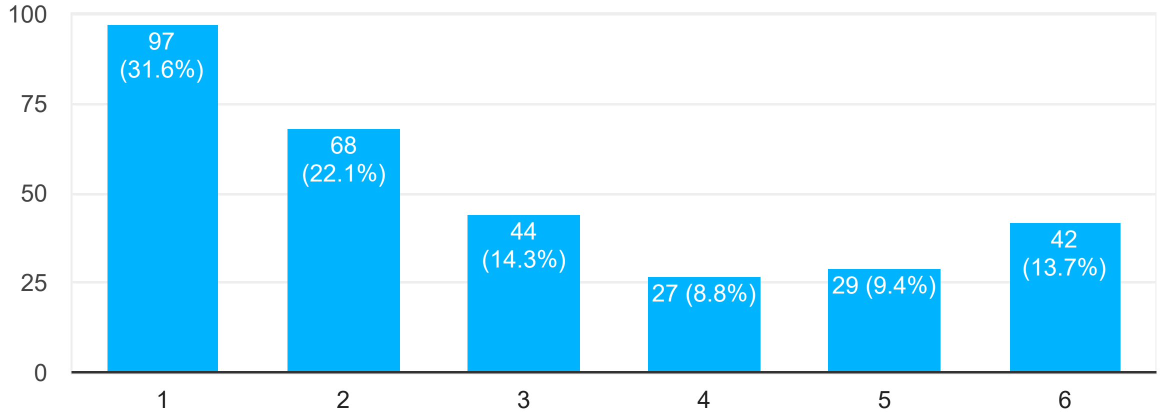

Discuss with coworkers about non-work issues ناقشت مع زملاء العمل قضايا لا تتعلق بالعمل

307 responses

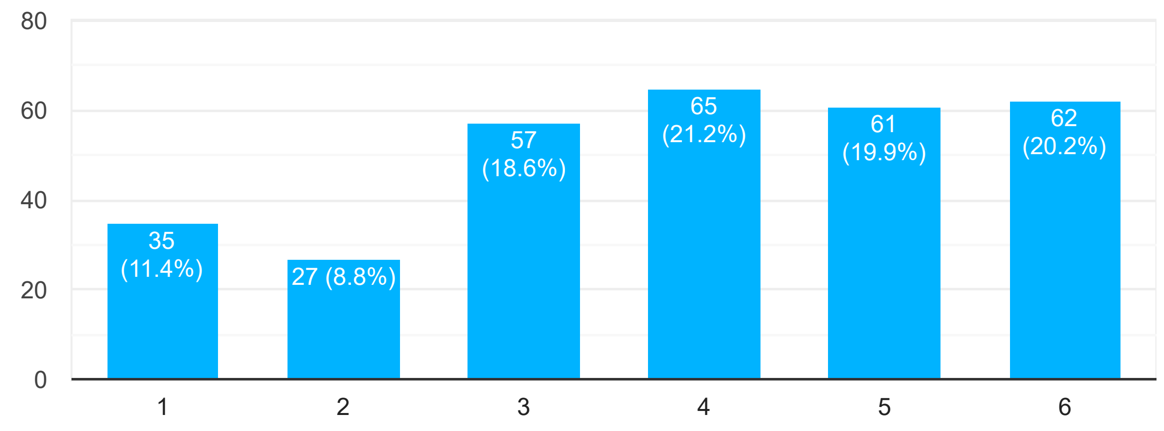

Thoughts of being absent التفكير في الغياب عن العمل

307 responses

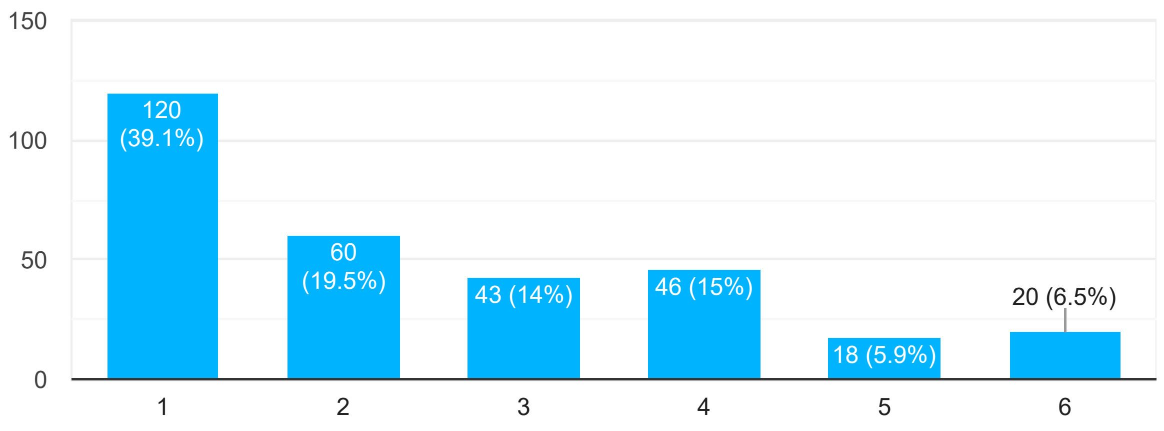

Left work station for unnecessary reasons ترك مكان العمل لأسباب غير ضرورية

307 responses

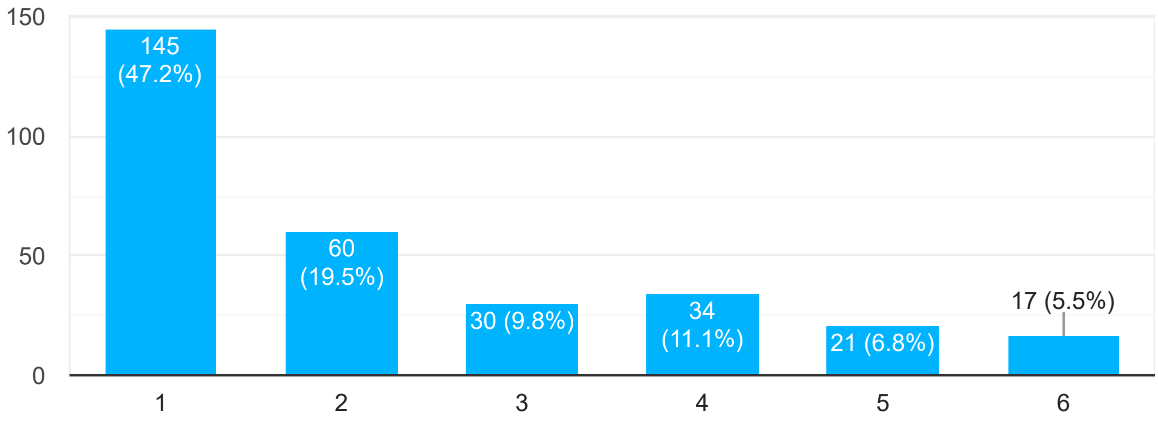

Section H

Coronavirus (COVID-19) event is a hindrance circumstance that completely interferes with my work and making difficult to achieve my work goals. Therefore, COVID-19 event appear to be a road block and impossible to overcome. .جائحة كرونا عائق يتداخل مع عملك ويمكن أن يقف في طريق قدرتك على تحقيق أهدافك وتبدو هذه الظروف وكأنها حاجز طريق، من المستحيل التغلب عليها ".

303 responses

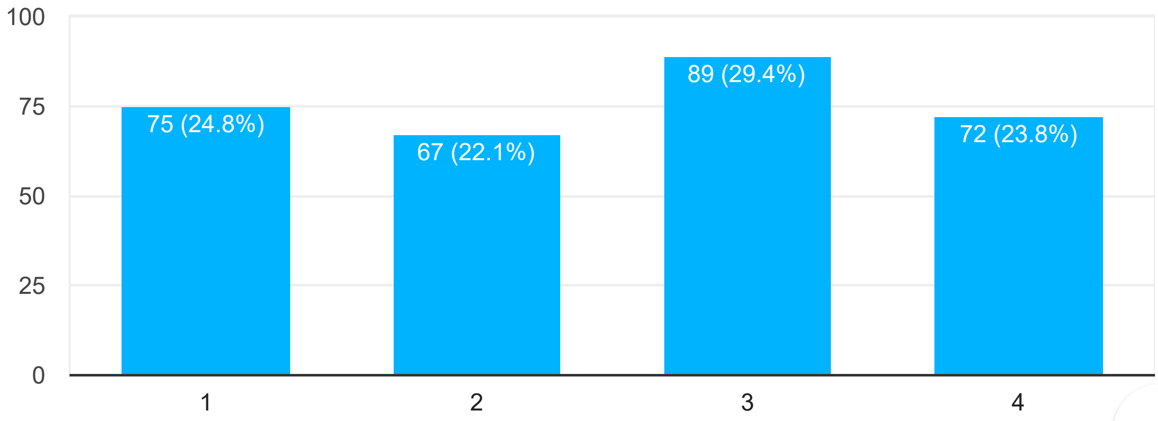

Based on these definitions, use the following scale to indicate your agreement or disagreement with each statement using a 4-point Likert Scale ranging from 1 = Strongly disagree to 4 = Strongly agree: Coronavirus (COVID-19) event is a challenging circumstance that although potentially stressful, I think I could overcome it. This is because COVID-19 event is motivating circumstance that can help me meet my work goals. بناءً على هذه التعريفات السابقة، استخدم المقياس ادناه للإشارة إلى موافقتك أو عدم موافقتك مع كل عبارة باستخدام مقياس ليكرت من 4 نقاط يبدأ من 1 = لا أوافق بشدة إلى 4 = أوافق بشدة: وتعد جائحة كرونا ظرفًا صعبًا ومرهقًا، وتعتقد أنه يمكنك التغلب عليه. هذا لأنه حدث محفز للظروف التي يمكن أن تساعدك على تحقيق أهداف عملك

299 responses

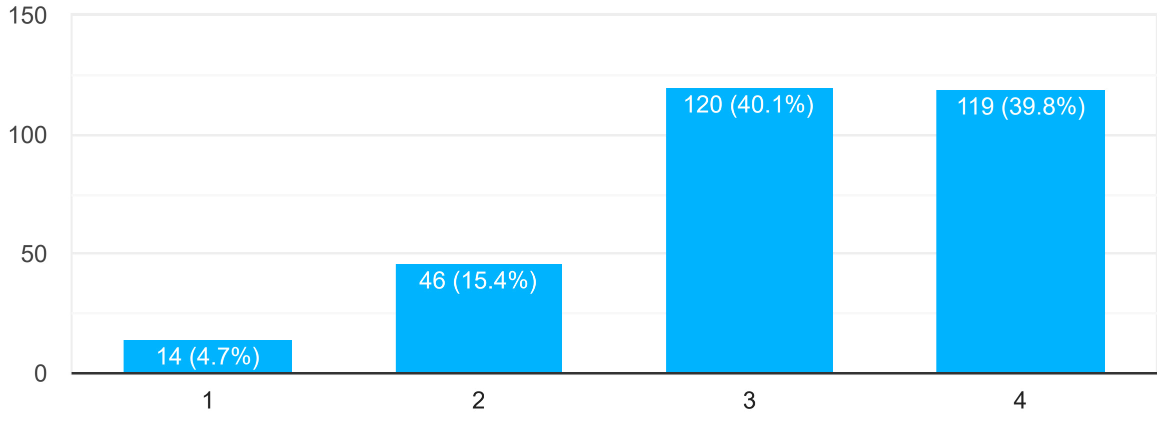

This content is neither created nor endorsed by Google. Report Abuse - Terms of Service - Privacy Policy

Google Forms
