# Supplementary material for: Survey data of coronavirus (COVID-19) thought concern, employees' work performance, employees background, feeling about job, work motivation, job satisfaction, psychological state of mind and family commitment in two middle east countries
Source: Data Brief. 2020 Dec 15;34:106661. doi: 10.1016/j.dib.2020.106661 (PMC7753928; doi:10.1016/j.dib.2020.106661)

“Analysis of Items Answers, COVID-19”

This file reflects each item (Question from 1 to 42) analysis with number and the percentage of responders from whole sample.

Figure 1. Gender

307 responses

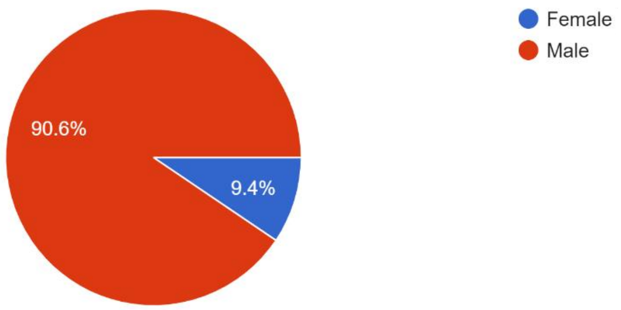

Figure 2. Marital Status

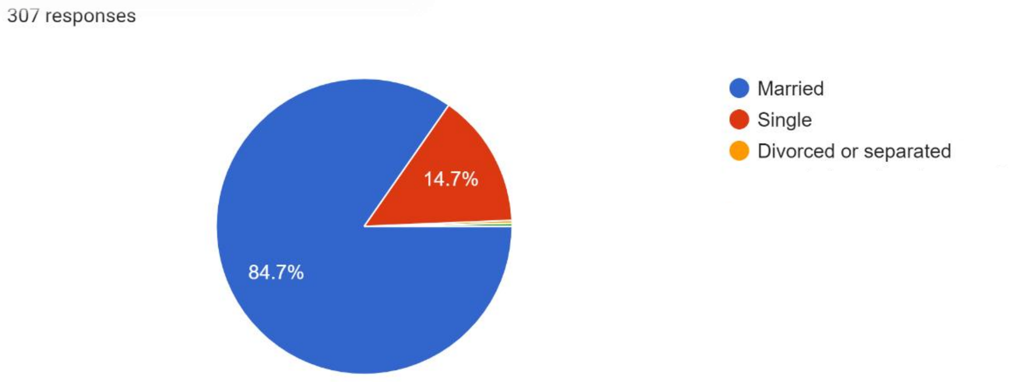

Figure 3. Age in Years

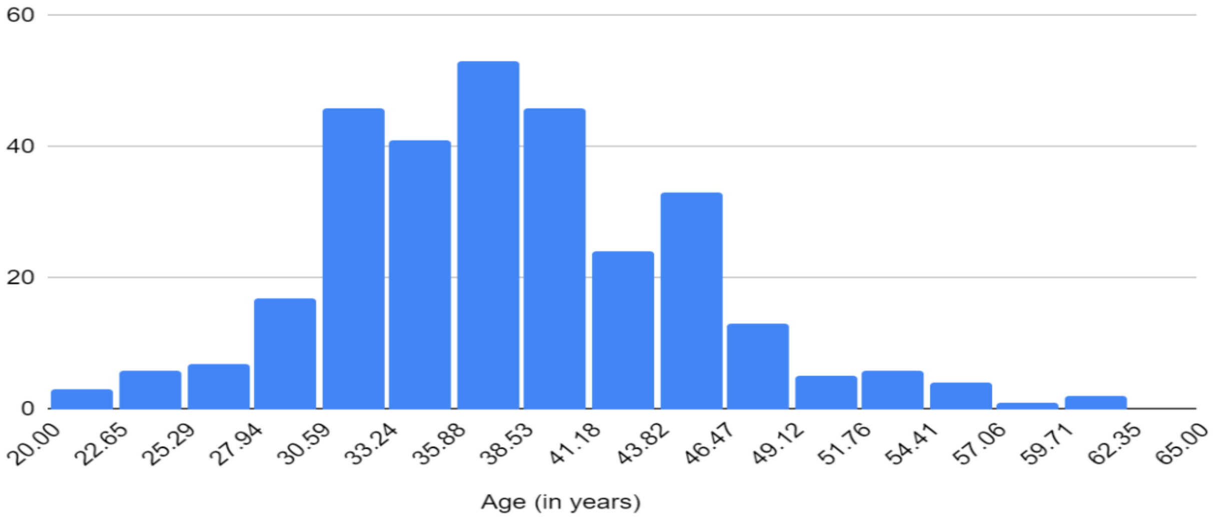

Figure 4. Employment Status

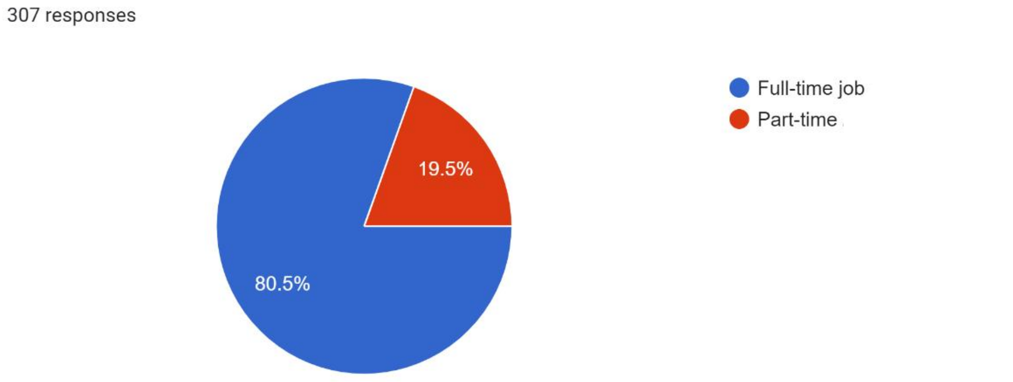

Figure 5. Level of Education

307 responses

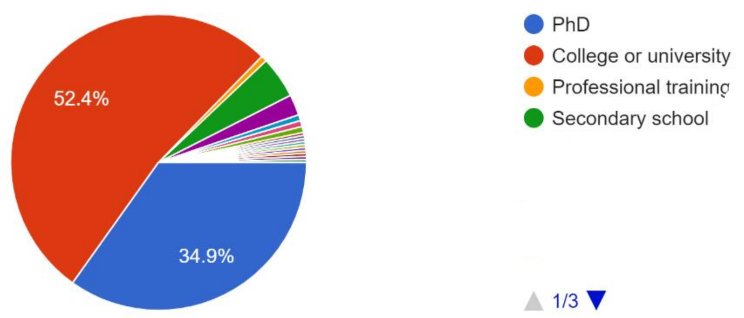

Figure 6. Organizational tenure (in years)

307 responses

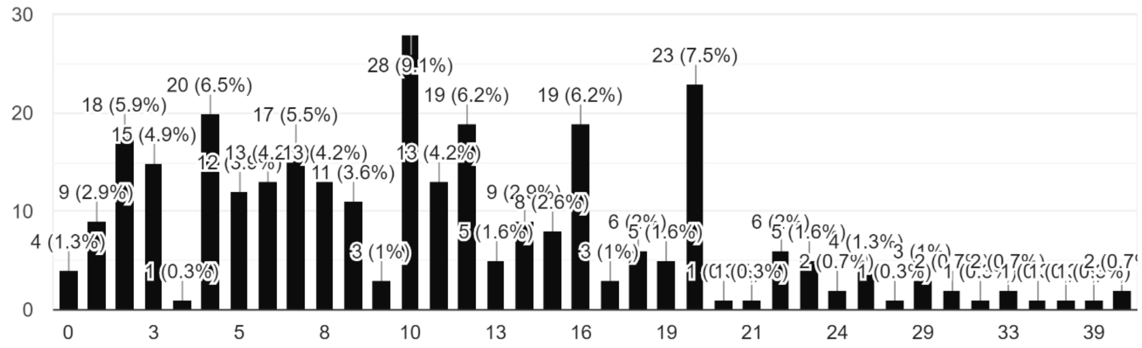

Figure 7. Despite the ease of restrictions in the past few weeks, I still feel distracted, or had to stay alert

307 responses

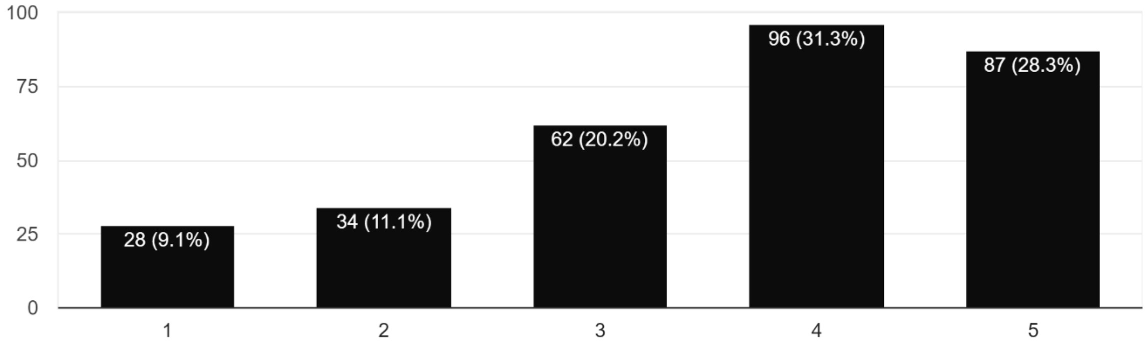

Figure 8. I physically get upset or annoyed by reminders of the coronavirus (COVID-19) event

307 responses

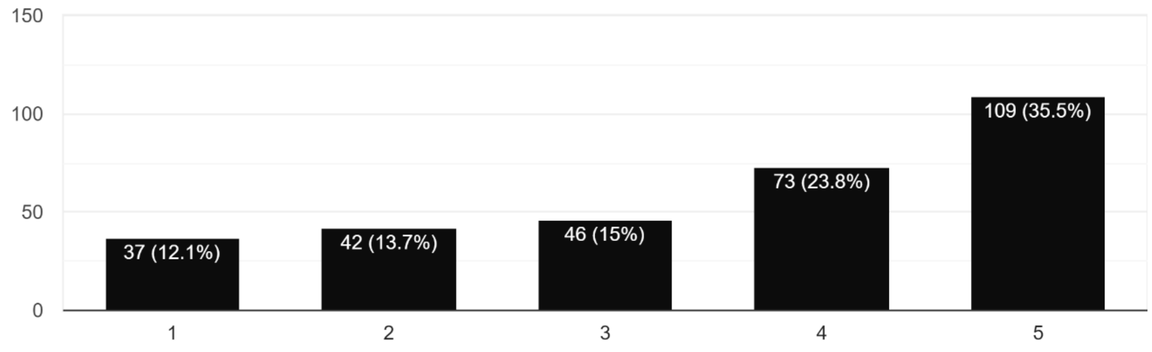

Figure 9. I still have difficulty concentrating since the recent COVID-19 pandemic crisis

307 responses

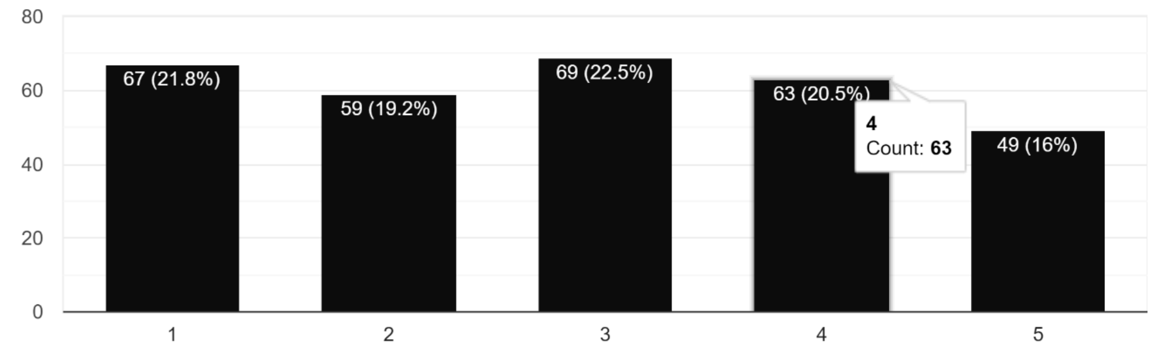

Figure 10. Despite the ease of restrictions in the past few weeks, I still have felt so distant or disconnected from other people

307 responses

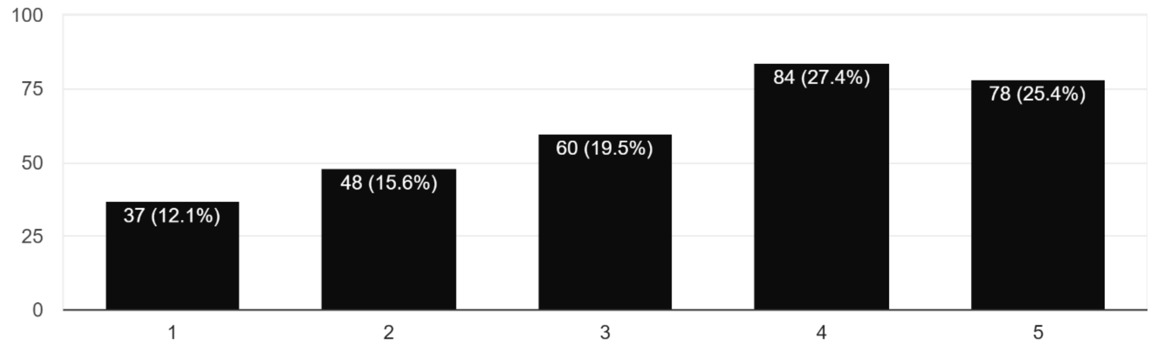

Figure 11. Watching coronavirus-related news and stories on traditional or social media, always make me feel as if the event is re-occurring.

307 responses

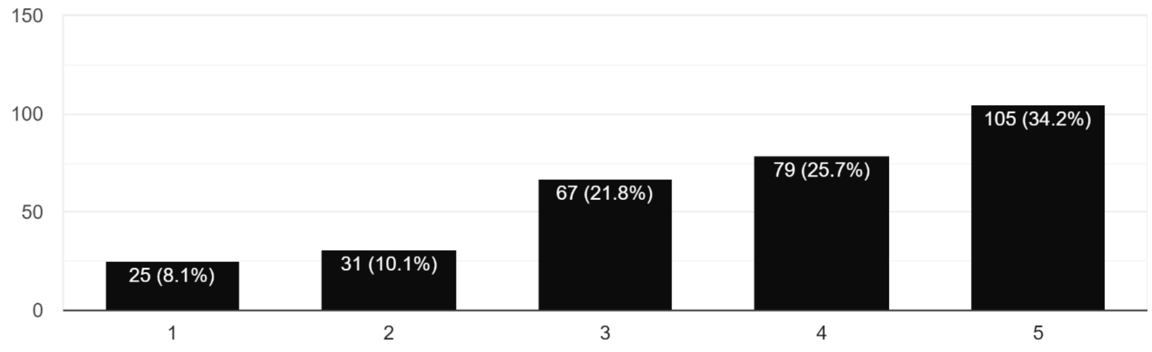

Figure 12. Despite the ease of restrictions in the past few weeks, I often have distressing dreams of the coronavirus (COVID-19) even.

307 responses

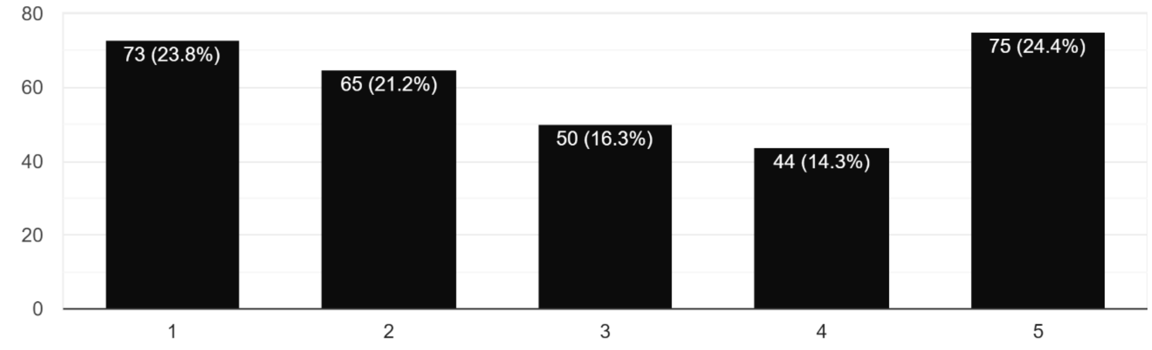

Figure 13. I am still avoiding things or going into situations which remind me about the coronavirus (COVID-19) event.

307 responses

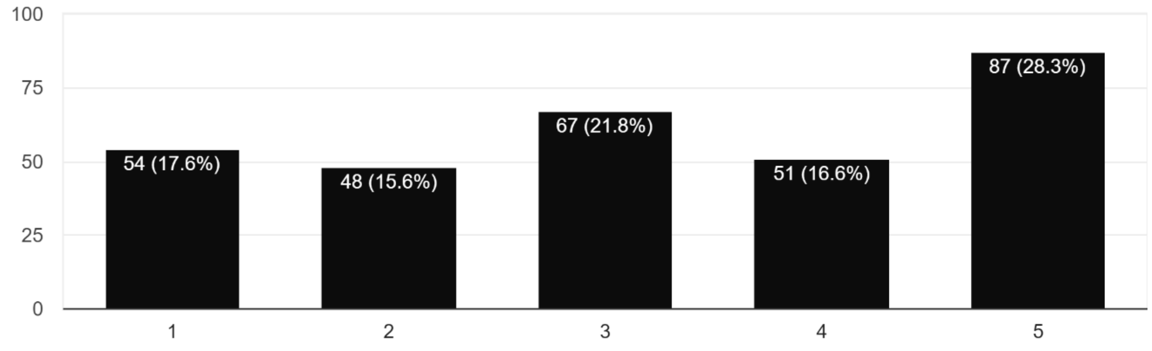

Figure 14. I still found myself unable to remember important parts of the coronavirus (COVID-19) event

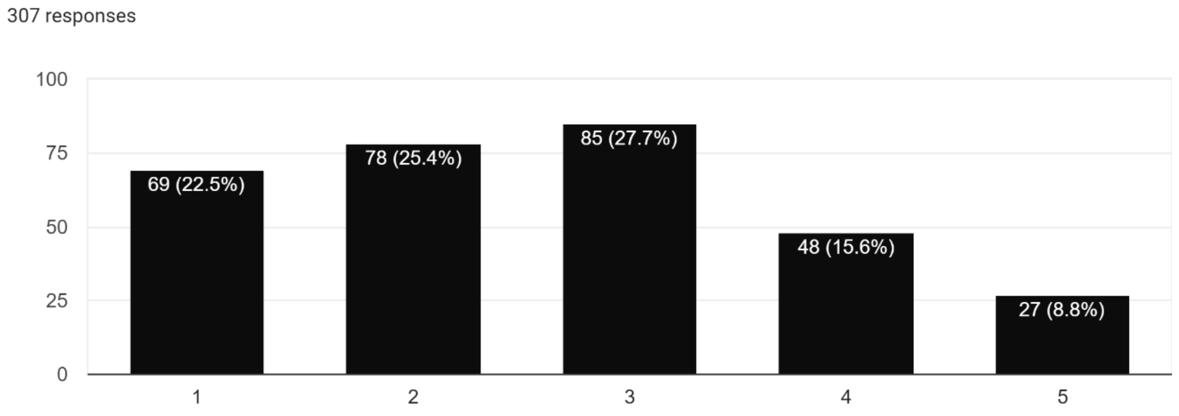

Figure 15. Despite the ease of restrictions in the past few weeks, I always find it difficult to fall asleep because I'm worrying about getting coronavirus (COVID-19.

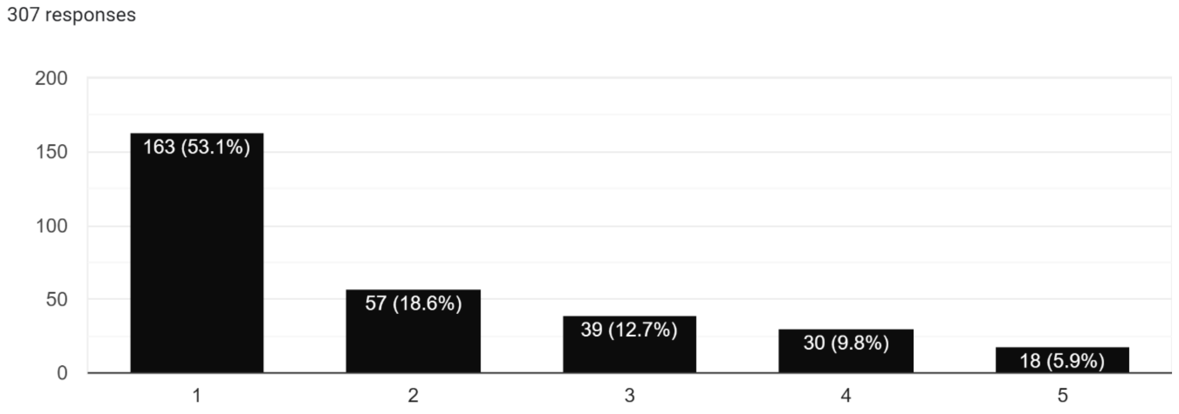

Figure 16. I consider my job rather unpleasant

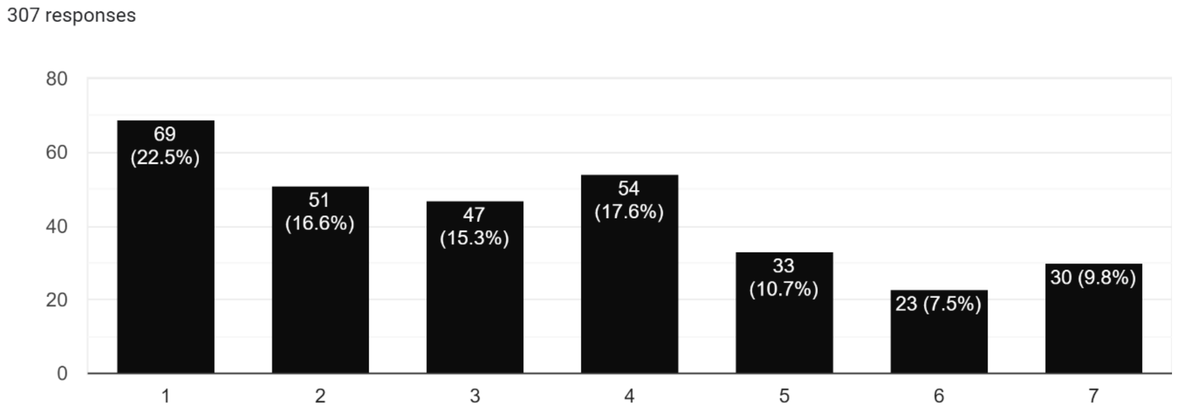

Figure 17. I find real enjoyment in my work

307 responses

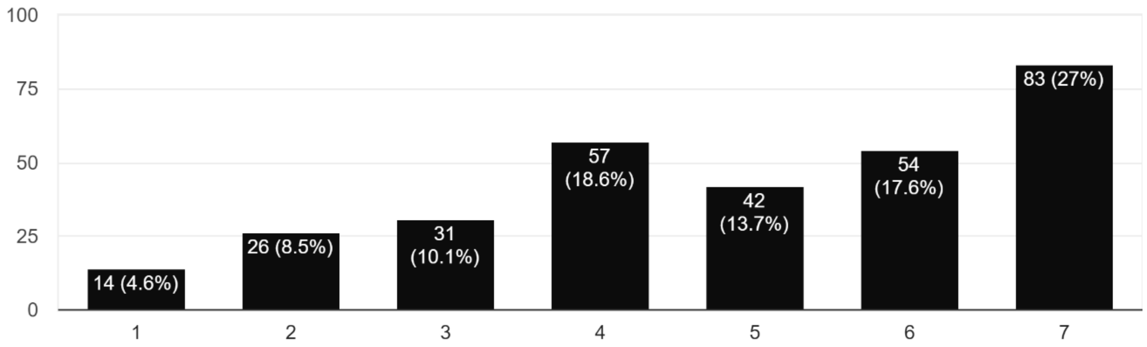

Figure 18. Each day of work seems like it will never end

307 responses

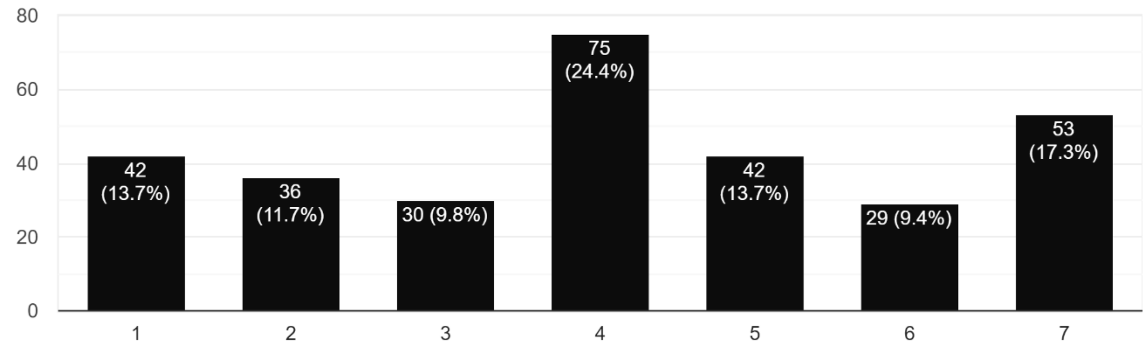

Figure 19. I feel fairly well satisfied with my present jol

307 responses

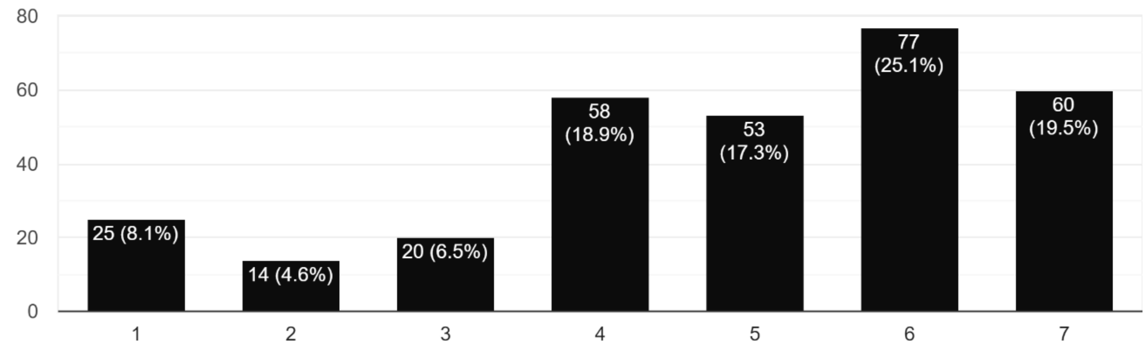

Figure 20. Most days I am enthusiastic about my work أنا متحمس في معظم الأيام لعملي

307 responses

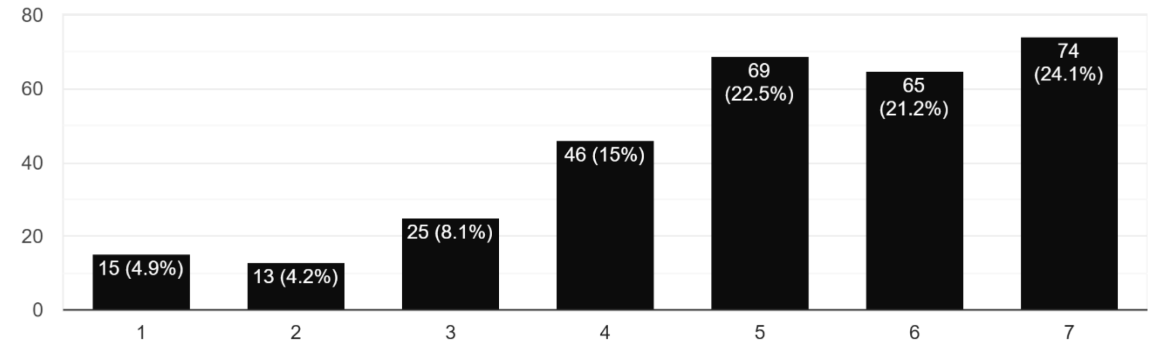

Figure 21. I always leave my tasks to the last minute.

307 responses

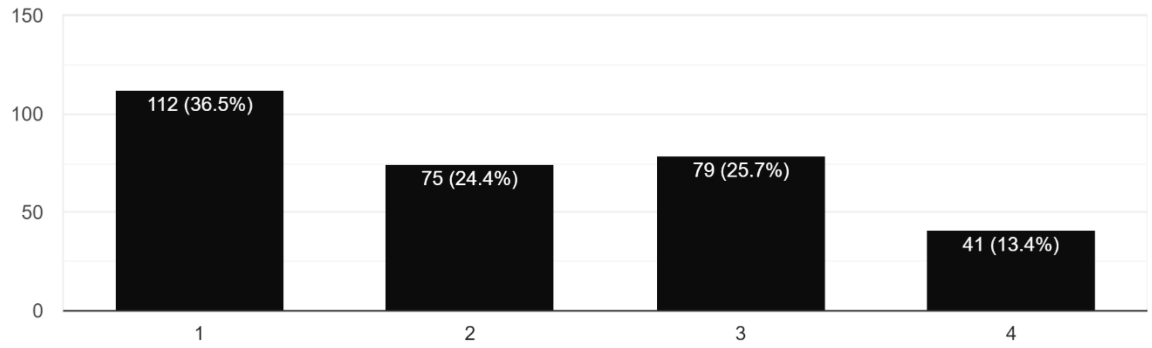

Figure 22. Sometimes, I feel disappointed with my performance at work, because I know I could have done better

307 responses

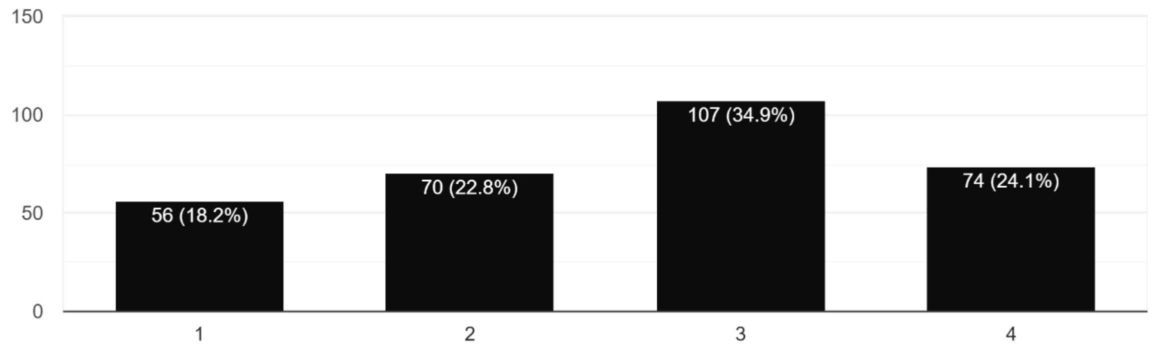

Figure 23. I consider myself a fundamental worker to the organization I work for, due to the high quality of my performance

307 responses

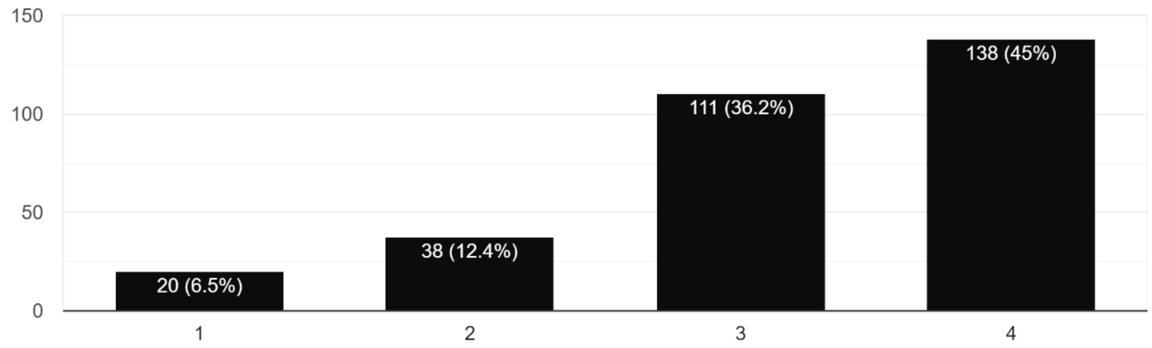

Figure 24. When I have a deadline to perform a certain task, I always finish it on time.

307 responses

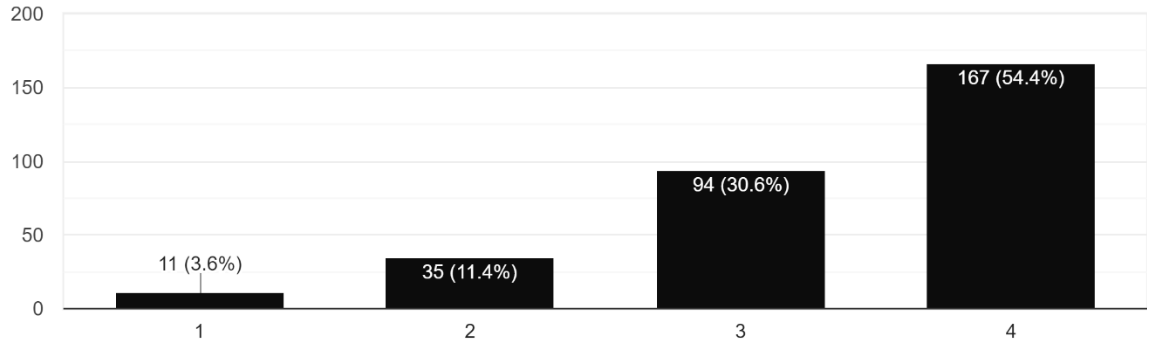

Figure 25. It is not always easy for me to perform tasks on time.

307 responses

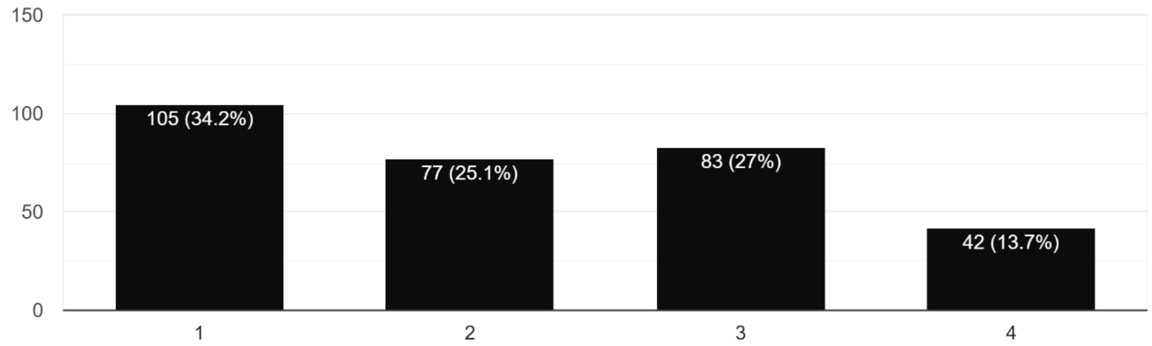

Figure 26. I was emotionally stable and sure of myself during the past few weeks.

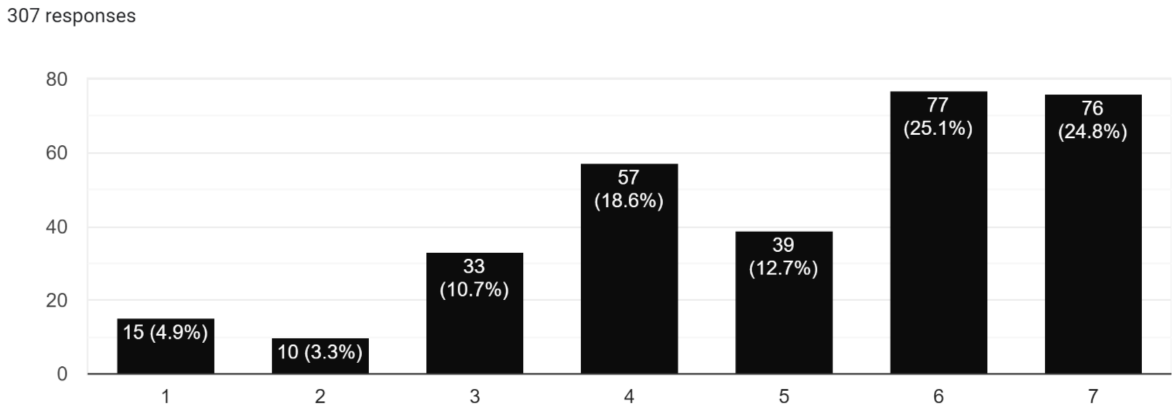

Figure 27. I felt cheerful, lighthearted during the past few weeks.

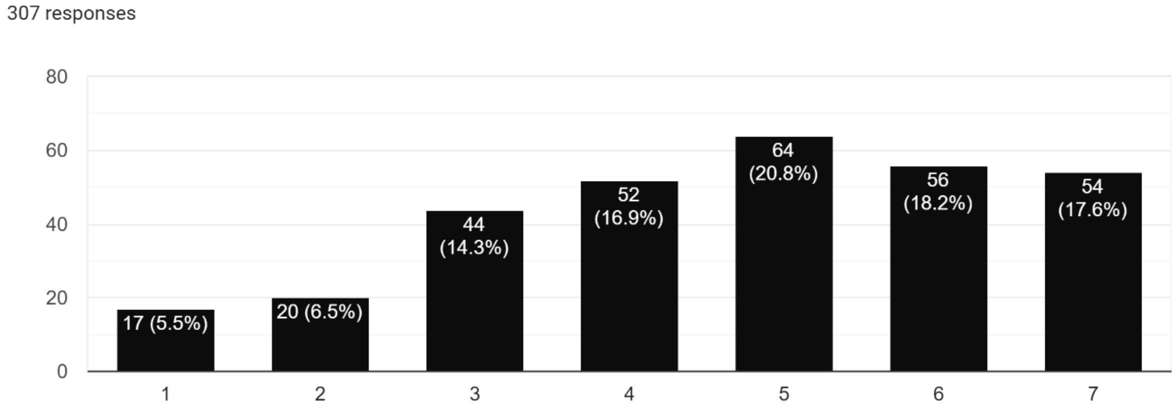

Figure 28. I felt tired, worn out, used up, or exhausted during the past few weeks.

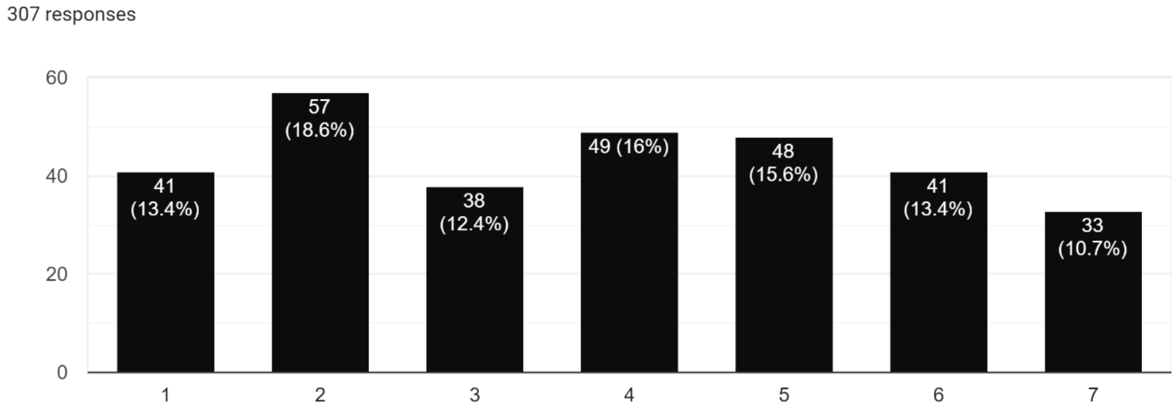

Figure 29.I felt bothered during the past few weeks

307 responses

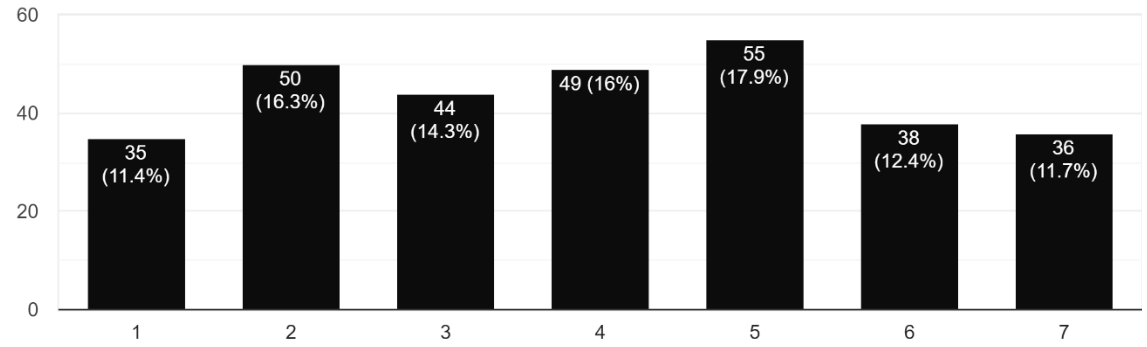

Figure 30.I felt downhearted and blue during the past few weeks

307 responses

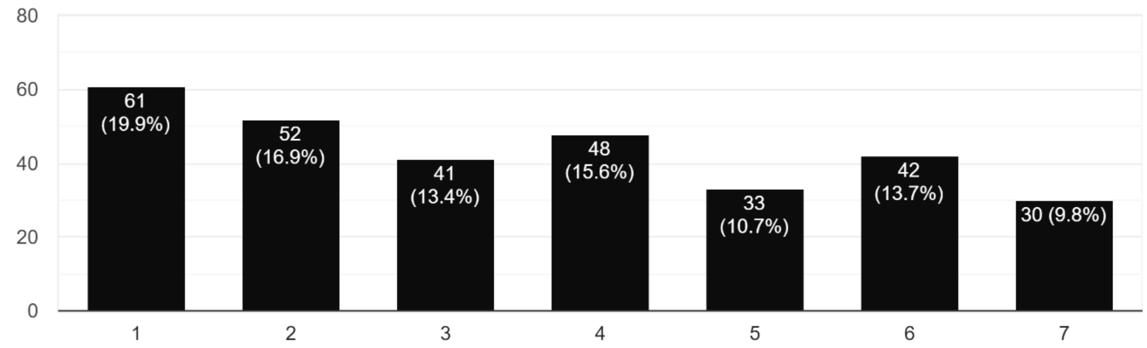

Figure 31.I felt much energy, pep, or vitality during the past few weeks شعرت بالكثير من الطاقة والحيوية والنشاط خلال الأسابيع القليلة الماضية

307 responses

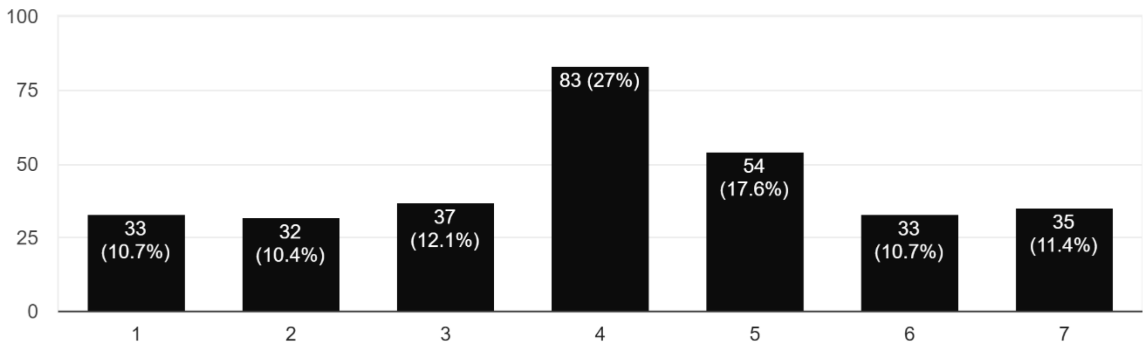

Figure 32. The time I spend on family responsibilities predominantly interferes with my work responsibilities.

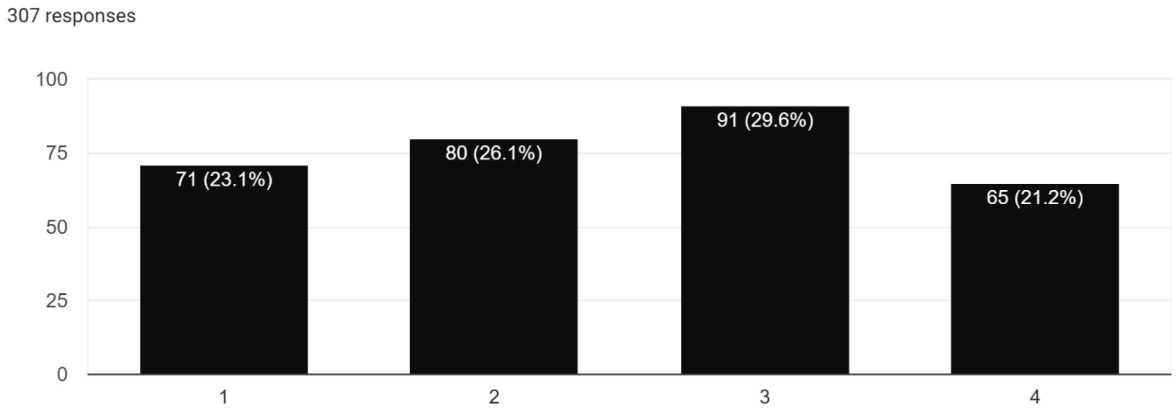

Figure 33. Spent work time on personal matters

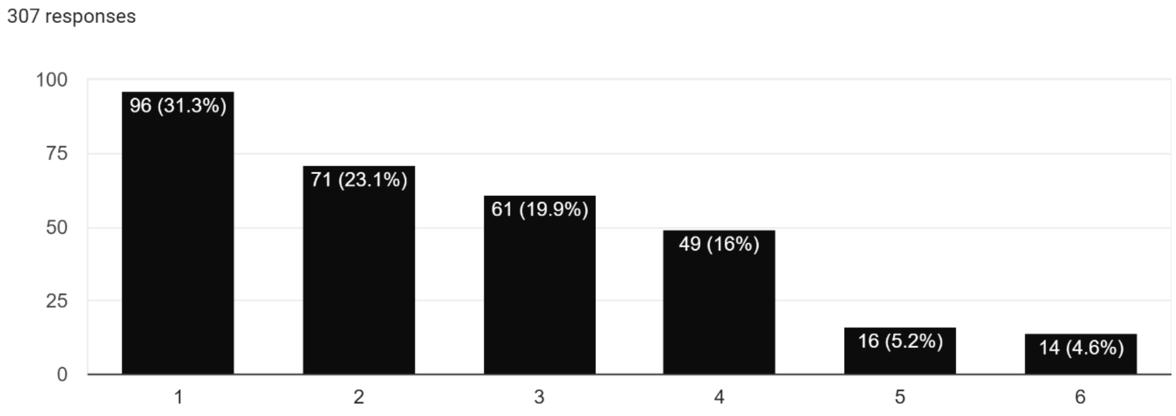

Figure 34. Put less effort into job than should have

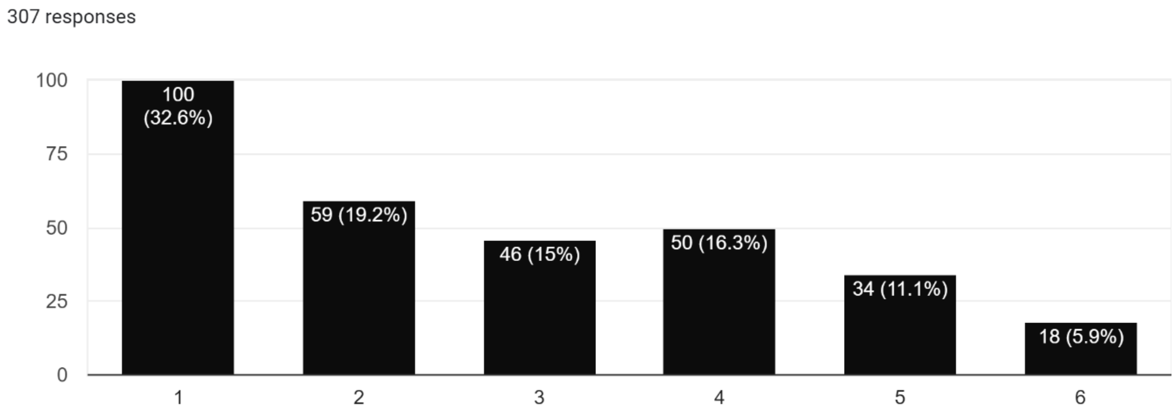

Figure 35.Let others do your work

307 responses

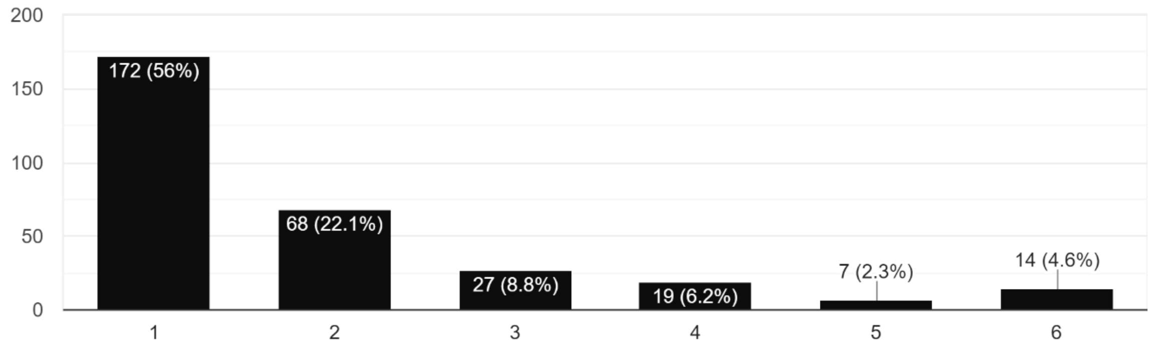

Figure 36. Daydreaming

307 responses

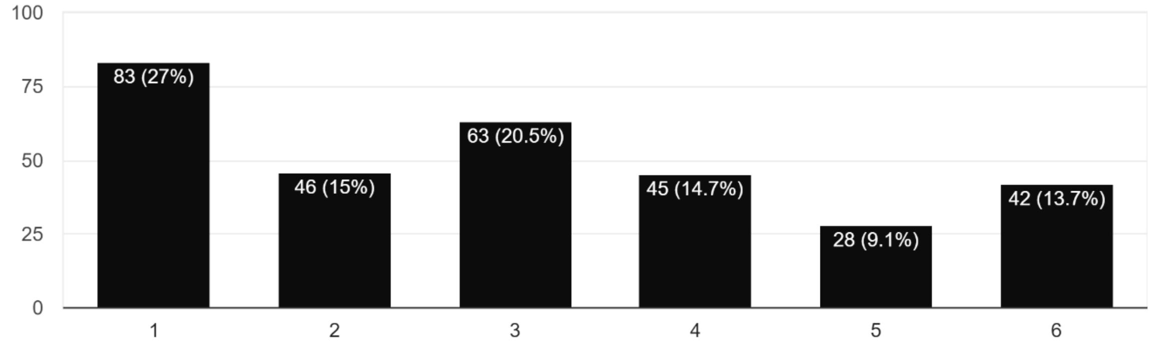

Figure 37.Thoughts of leaving current job

307 responses

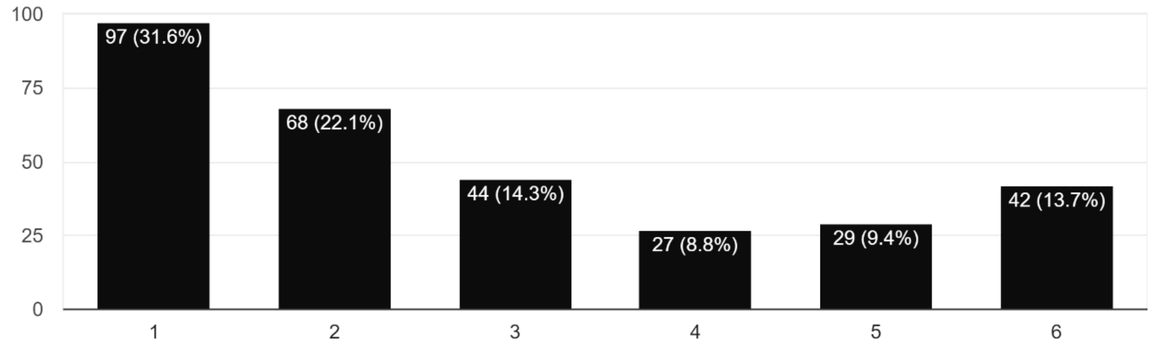

Figure 38. Discuss with coworkers about non-work issues

307 responses

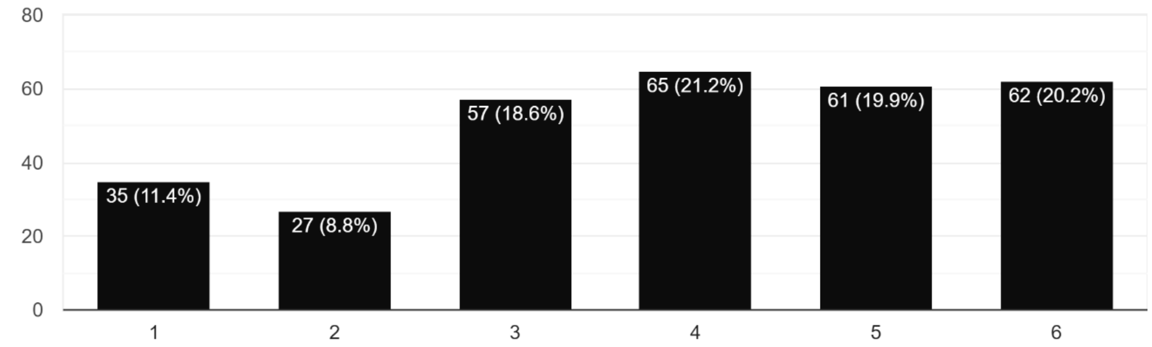

Figure 39. Thoughts of being absent

307 responses

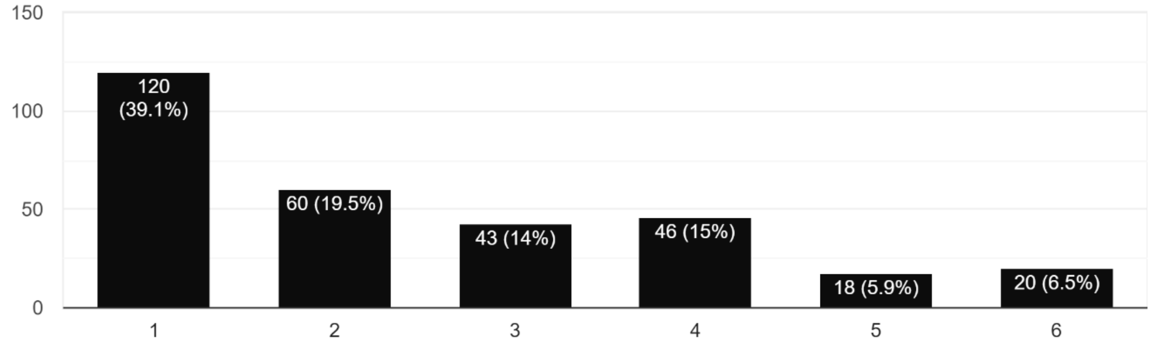

Figure 40. Left work station for unnecessary reasons

307 responses

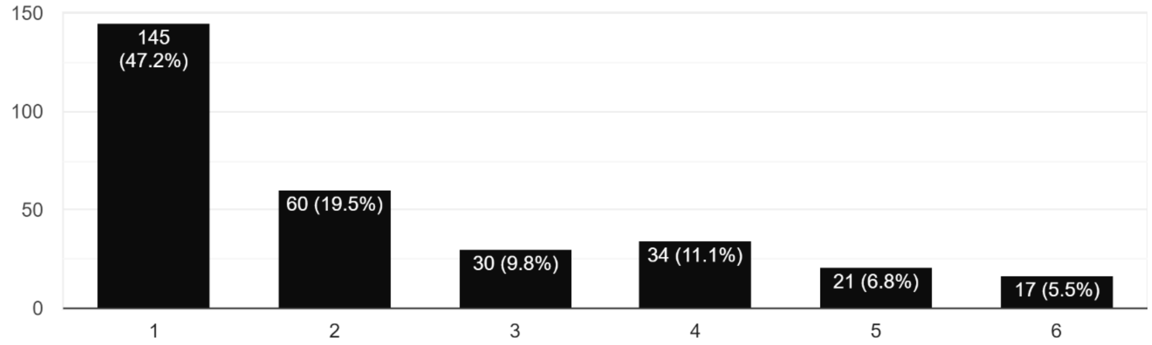

Figure 41. Coronavirus (COVID-19) event is a hindrance circumstance that completely interferes with my work and making difficult to achieve my work goals. Ther..

303 responses

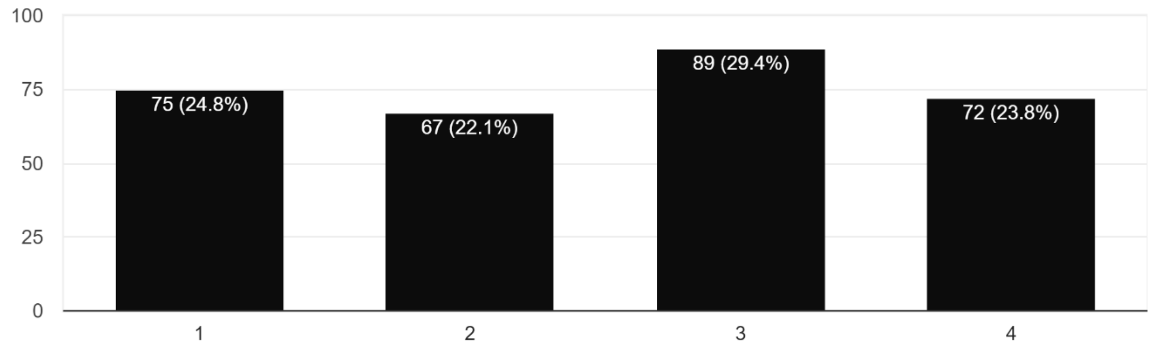

Figure 42. Based on these definitions, use the following scale to indicate your agreement or disagreement with each statement using a 4-point Likert Scale rangin.

299 responses

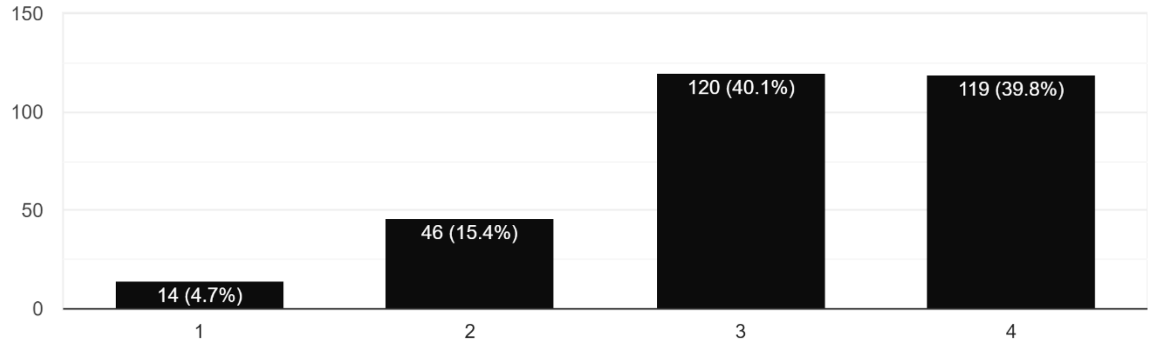

Supplement: Supplementary file 4 [file mmc4.pdf]
